# Human *GPR17* missense variants identified in metabolic disease patients have distinct downstream signaling profiles

**DOI:** 10.1016/j.jbc.2021.100881

**Published:** 2021-06-16

**Authors:** Jason M. Conley, Hongmao Sun, Kristin L. Ayers, Hu Zhu, Rong Chen, Min Shen, Matthew D. Hall, Hongxia Ren

**Affiliations:** 1 Herman B. Wells Center for Pediatric Research, Department of Pediatrics, Indiana University School of Medicine, Indianapolis, Indiana, USA; 2 Center for Diabetes and Metabolic Diseases, Indiana University School of Medicine, Indianapolis, Indiana, USA; 3 National Center for Advancing Translational Sciences, National Institutes of Health, Rockville, Maryland, USA; 4 Department of Genetics and Genomic Sciences, The Icahn Institute for Genomics and Multiscale Biology, Icahn School of Medicine at Mount Sinai, New York, New York, USA; 5 Sema4, a Mount Sinai venture, Stamford, Connecticut, USA; 6 Department of Pharmacology & Toxicology, Indiana University School of Medicine, Indianapolis, Indiana, USA; 7 Stark Neurosciences Research Institute, Indiana University School of Medicine, Indianapolis, Indiana, USA; 8 Department of Biochemistry & Molecular Biology, Indiana University School of Medicine, Indianapolis, Indiana, USA; 9 Department of Anatomy, Cell Biology & Physiology, Indiana University School of Medicine, Indianapolis, Indiana, USA

**Keywords:** metabolism, human genetics, G-protein-coupled receptor (GPCR), signal transduction, metabolic disease, gene regulation, signaling, cyclic AMP (cAMP), calcium, arrestin, AgRP, agouti-related peptide, FoxO1, Forkhead box protein O1, GPCR, G-protein-coupled receptor, HA, hemagglutinin, LDL, low-density lipoprotein, PBS, phosphate-buffered saline, POMC, proopiomelanocortin, SIR, severe insulin resistance

## Abstract

GPR17 is a G-protein-coupled receptor (GPCR) implicated in the regulation of glucose metabolism and energy homeostasis. Such evidence is primarily drawn from mouse knockout studies and suggests GPR17 as a potential novel therapeutic target for the treatment of metabolic diseases. However, links between human *GPR17* genetic variants, downstream cellular signaling, and metabolic diseases have yet to be reported. Here, we analyzed *GPR17* coding sequences from control and disease cohorts consisting of individuals with adverse clinical metabolic deficits including severe insulin resistance, hypercholesterolemia, and obesity. We identified 18 nonsynonymous *GPR17* variants, including eight variants that were exclusive to the disease cohort. We characterized the protein expression levels, membrane localization, and downstream signaling profiles of nine GPR17 variants (F43L, V96M, V103M, D105N, A131T, G136S, R248Q, R301H, and G354V). These nine GPR17 variants had similar protein expression and subcellular localization as wild-type GPR17; however, they showed diverse downstream signaling profiles. GPR17-G136S lost the capacity for agonist-mediated cAMP, Ca^2+^, and β-arrestin signaling. GPR17-V96M retained cAMP inhibition similar to GPR17-WT, but showed impaired Ca^2+^ and β-arrestin signaling. GPR17-D105N displayed impaired cAMP and Ca^2+^ signaling, but unaffected agonist-stimulated β-arrestin recruitment. The identification and functional profiling of naturally occurring human GPR17 variants from individuals with metabolic diseases revealed receptor variants with diverse signaling profiles, including differential signaling perturbations that resulted in GPCR signaling bias. Our findings provide a framework for structure–function relationship studies of GPR17 signaling and metabolic disease.

Metabolic diseases including obesity, type 2 diabetes, and cardiovascular diseases are worldwide health challenges due to the increasing prevalence and negative effects on quality of life, premature mortality, and increased healthcare burden (1, 2). Identifying new therapeutic targets may fulfill the need to develop safer and more effective pharmacological treatment strategies to combat metabolic diseases. G-protein-coupled receptors (GPCRs) are a family of seven transmembrane domain cell-surface receptor proteins that are of particular interest, as they regulate metabolic homeostasis by transducing metabolic signals, in the form of hormones and metabolites, into cellular signaling events (3). Moreover, GPCRs are amenable to pharmacological manipulation, as approximately one-third of all United States Food and Drug Administration (FDA)-approved drugs target GPCRs (4). While some GPCRs have been successfully exploited for treatment of metabolic diseases (5), much of the GPCRome is understudied (6, 7) and thus warrants more mechanistic and preclinical studies for GPCRs with metabolic implications.

GPR17 is such an understudied GPCR that regulates glucose metabolism and energy homeostasis in genetic mouse models. We and others have identified GPR17 as an effector of the transcription factor, Forkhead box protein O1 (FoxO1) (8, 9). In the hypothalamus, FoxO1 impinges on both insulin and leptin signaling pathways (10), and FoxO1 genetic ablation dramatically downregulates Gpr17 expression in agouti-related peptide (AgRP) neurons (8). Consistent with the observation that transcriptional regulation of Gpr17 expression is mediated by FoxO1, hypothalamic Gpr17 expression is also modulated by nutritional changes such as fasting and refeeding, where hypothalamic Gpr17 expression during fasting is higher than in the refeeding condition (8). Subsequent study of Gpr17 in AgRP neurons demonstrated that deletion of Gpr17 in this neuron type decreases food intake, increases relative energy expenditure, increases satiety, and increases sensitivity to circulating insulin and leptin (11). Gpr17 knockout in another hypothalamic neuron type implicated in feeding behavior, proopiomelanocortin (POMC) neurons, provides protection from high-fat-diet-induced weight gain in mice (12). Studies from other groups also showed that oligodendrocyte-specific Gpr17 knockout protects mice from high-fat-diet-induced metabolic defects (13), though whole-body germline GPR17 knockout studies yielded inconsistent results (13, 14). Taken together, the rodent tissue-specific knockout studies suggest that GPR17 contributes to metabolic regulation, likely in a cell-type-specific manner.

In contrast to the animal studies, the molecular signaling mechanisms of GPR17 are less clear. GPR17 was first identified as a novel orphan GPCR in 1998 by screening a human hippocampus cDNA library with a probe for purinergic P2Y-receptor homologous sequences (15). Human GPR17 has two isoforms (*i.e.*, long and short), while mouse Gpr17 only has one isoform that is more similar to the human short isoform (16). The human GPR17 long isoform (hGPR17L) differs from the short isoform by having an extra 28 amino acids at the extracellular N-terminus. Consistent with a phylogenetic position of GPR17 between cysteinyl leukotriene receptor and purinergic receptor clusters (17), an initial deorphanization study demonstrated that GPR17 functionally responds to cysteinyl leukotrienes and uracil nucleotides (18). In contrast, subsequent reports directly disputed that cysteinyl leukotrienes and uracil nucleotides are the endogenous ligands for GPR17 (19, 20). Despite continuing uncertainty surrounding the endogenous GPR17 ligand(s), the synthetic small molecule agonist MDL29,951 has facilitated the understanding of G protein coupling and downstream signaling pathways (19, 20, 21, 22). MDL29,951-stimulated GPR17 signaling modulates cAMP and Ca^2+^ signaling pathways by way of Gαi/o and Gαq-coupling in cells with heterologous expression of GPR17 (19, 20, 21). Interestingly, there is also evidence for Gαs- (19), Gα12/13- (21), and β-arrestin-mediated GPR17 signaling (19, 22), suggesting the potential for diverse downstream signaling outcomes that are dependent on the cellular context of signaling proteins in different cell types.

Genetic mouse models are indispensable for understanding diabetes and obesity pathophysiology (23), and previous studies in our laboratory and other groups demonstrated the role of Gpr17 in metabolic regulation (8, 11, 12, 13). However, human GPR17 genetic studies have yet to be reported. Human genetic approaches, including the identification and study of natural missense genetic variants, offer opportunities to gain novel insight into the contribution of genetic variation to clinical phenotypes associated with metabolic diseases and related traits (24). Such genetic approaches can also be used to assess novel therapeutic target potential and guide target development (24). For example, recent studies of functional signaling consequences of GPCR missense variants suggest the possibility that variant-associated pharmacological signaling properties may contribute to altered risk of disease outcomes (25) and therapeutic responses (26). These studies highlight the utility of understanding the functional signaling consequences of GPCR variants. The present study was designed to identify GPR17 variants in human populations and to characterize their functional signaling properties. We report the identification of naturally occurring missense variants of *GPR17* and the functional signaling profiles of nine variants identified in individuals with metabolic disease.

## Results

### Sequence analysis identified human GPR17 genetic variants in control and metabolic disease cohorts

**Figure 1:**
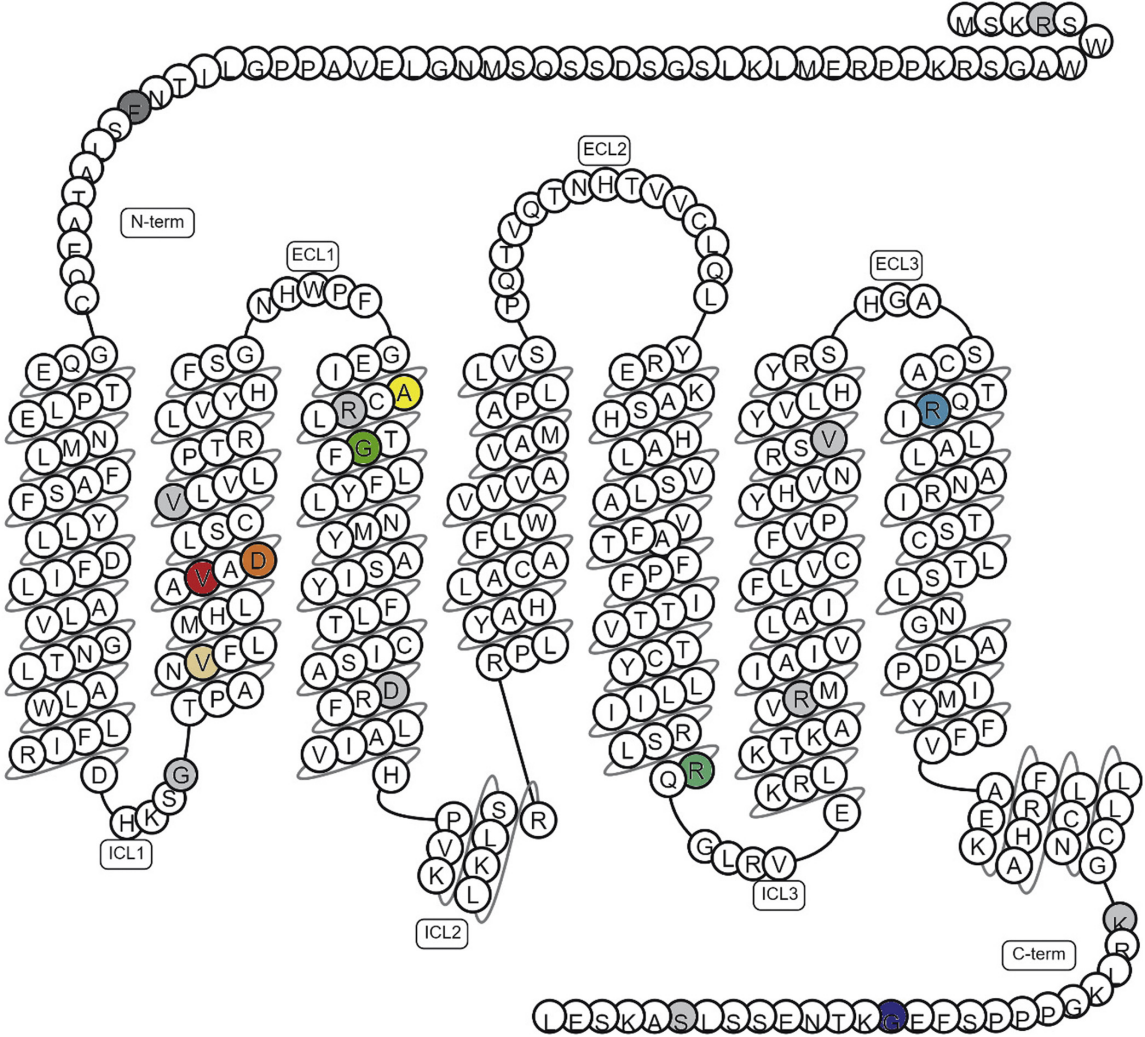
**Schematic representation of the human GPR17 long isoform denoting nonsynonymous amino acid variants.** Nonsynonymous amino acid variants characterized here are shaded according to the following coloring scheme. *Dark gray* for F43; *beige* for V96; *red* for V103; *orange* for D105; *yellow* for A131; *green* for G136; *turquoise* for R248; *teal* for R301; *blue* for G354. Other GPR17 variants identified are shaded in *light gray*. ECL, extracellular loop; ICL, intracellular loop; N-term, N terminus; C-term, C terminus. Schematic was generated by gpcrdb.org.

Human *GPR17* gene sequences were obtained and analyzed for 4054 samples from control and metabolic disease cohorts from the UK10K project. The control cohorts consisted of 2432 individuals from the Avon Longitudinal Study of Parents and Children (ALSPAC; 740 samples) and The Department of Twin Research and Genetic Epidemiology Twin Registry at King's College London (TWINS; 1692 samples). The metabolic disease cohort was a collection of patients diagnosed with severe insulin resistance (SIR; 125 samples), hypercholesterolemia (HYPERCHOL; 125 samples), and obesity (1372 samples). The patients with obesity were from the Severe Childhood Onset Obesity Project (OB_SCOOP; 961 samples) and obese individuals from the Generation Scotland study (OB_GS; 411 samples). Across control and disease cohorts, 18 nonsynonymous *GPR17* genetic variants were identified (Table 1). The rarity of these variants, each with frequencies <0.5% in the general population from the Genome Aggregation Database (gnomAD), limits the meaningful statistical interpretation. Topologically, variants were distributed throughout the receptor in regions that are expected to be extracellular, transmembrane helices, and intracellular segments of the protein (Fig. 1). Of the 18 missense *GPR17* variants identified, eight (*i.e.*, V96M, V103M, D105N, A131T, G136S, R248Q, R301H, and G354V) were exclusive to the disease cohorts, and one (*i.e.*, F43L) occurred more frequently in the disease cohort than the control cohort (odds ratio 2.4, *p* = 0.013) and in obese individuals *versus* nonobese individuals (odds ratio 4.9, *p* = 0.022). Here, we sought to experimentally characterize the expression, localization, and functional signaling consequences of the naturally occurring human GPR17 genetic variants observed in the metabolic disease cohorts.

**Table 1:** Summary of nonsynonymous human *GPR17* variants identified in UK10K cohorts

SNP	AA change	ALSPAC AF (AC)	TWINS AF (AC)	Control (AC)	HYPERCHOL AF (AC)	SIR AF (AC)	OB SCOOP AF (AC)	OB GS AF (AC)	Metabolic disease (AC)
2_128407596_C_T	R4W	0.0007 (1)	0.0012 (4)	5	0	0	0	0	0
2_128408354_C_A	F43L	0.0068 (10)	0.0015 (5)	15	0	0	0.007 (14)	0.0073 (6)	20
2_128408496_G_A	G91R	0.0007 (1)	0	1	0	0	0	0	0
2_128408511_G_A	V96M	0	0	0	0	0	0	0.0012 (1)	1
2_128408532_G_A	V103M	0	0	0	0	0	0.0005 (1)	0	1
2_128408538_G_A	D105N	0	0	0	0.008 (2)	0	0	0.0012 (1)	3
2_128408550_G_A	V109M	0.0014 (2)	0.0006 (2)	4	0	0.004 (1)	0.0005 (1)	0.0012 (1)	3
2_128408616_G_A	A131T	0	0	0	0	0	0.0005 (1)	0	1
2_128408622_C_T	R133C	0.0020 (3)	0.0012 (4)	7	0	0	0.0016 (3)	0.0012 (1)	4
2_128408631_G_A	G136S	0	0	0	0	0	0	0.0012 (1)	1
2_128408691_G_T	D156Y	0	0.0003 (1)	1	0	0	0	0	0
2_128408968_G_A	R248Q	0	0	0	0	0	0.0005 (1)	0	1
2_128409013_G_A	R263H	0.0007 (1)	0.0012 (4)	5	0	0	0	0.0012 (1)	1
2_128409078_G_A	V285I	0	0.0003 (1)	1	0	0	0.0005 (1)	0	1
2_128409127_G_A	R301H	0	0	0	0.004 (1)	0	0.0005 (1)	0	2
2_128409256_G_T	R344M	0.0007 (1)	0	1	0	0	0	0	0
2_128409286_G_T	G354V	0	0	0	0.004 (1)	0	0	0	1
2_128409309_A_G	S362G	0.0007 (1)	0	1	0	0	0	0	0

AA, amino acid; AC, allele count; AF, allele frequency; ALSPAC, Avon Longitudinal Study of Parents and Children sample set; HYPERCHOL, hypercholesterolemia sample set; OB GS, Generation Scotland obesity sample set; OB SCOOP, Severe Childhood Onset Obesity Project sample set; SIR, severe insulin resistance sample set; SNP, single nucleotide polymorphism; TWINS, The Department of Twin Research and Genetic Epidemiology Twin Registry at King's College London sample set.

### Characterization of human GPR17 expression level and localization

**Figure 2:**
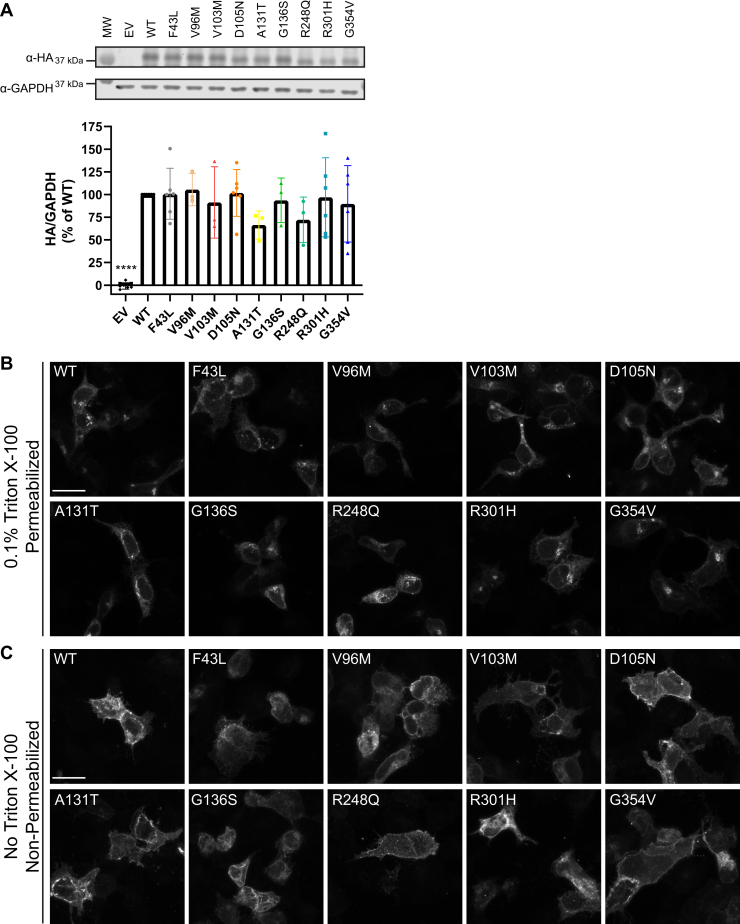
**Human GPR17L variant expression and localization.***A*, representative western blot (*upper panel*) and quantification (*lower panel*) of transient expression of pcDNA3.1(−) empty vector, hGPR17L-WT, or hGPR17L variants in HEK293 cells. Data represent mean ± SD of western blots for lysate from three to six independent transfections and were analyzed by one-sample *t* test compared with 100. ∗∗∗∗*p* < 0.0001. Representative images of total GPR17 (*i.e.*, 0.1% Triton X-100 permeabilized. Scale bar, 25 μm. Pixel intensity range, 0–65,535) (*B*) or cell surface GPR17 (*i.e.*, nonpermeabilized. Scale bar, 25 μm. Pixel intensity range, 0–30,000) (*C*) immunostaining in HEK293 cells transiently transfected with pcDNA3-HA-hGPR17L or pcDNA3-HA-hGPR17L-variants. EV, empty vector.

In order to assess if the nonsynonymous hGPR17L variants have defects in protein expression and trafficking, we quantitively measured the protein levels and localization of nine metabolic-disease-related variants expressed. The expression levels of hGPR17L variants were characterized by western blotting with an α-hemagglutinin (HA) antibody for transiently transfected N-terminally HA-epitope-tagged hGPR17L in HEK293 cells. Immunoreactivity was detected for all hGPR17L variants, but not empty vector transfected cells (Fig. 2*A*, upper panel). Furthermore, quantification of the immunoreactive bands showed that there was no statistically significant difference of protein expression level between the hGPR17L variants and wild-type hGPR17L (Fig. 2*A*, lower panel). Receptor localization was evaluated by immunostaining N-terminally HA-tagged hGPR17L transiently transfected in HEK293 cells. Both cell surface and cytoplasmic receptor was detected under the 0.1% Triton X-100 permeabilization condition (Fig. 2*B*), while receptor localized on plasma membrane was detected in nonpermeabilized cells (Fig. 2*C*). No staining was observed in empty vector transfected cells (data not shown). All hGPR17L variants displayed similar cellular localization patterns as compared with hGPR17L-WT (Fig. 2*B*). hGPR17L-WT and hGPR17L variants had similar levels of cell surface staining (Fig. 2, *B* and *C*). Quantification of 0.1% Triton X-100 permeabilized (Fig. S1*A*) and nonpermeabilized (Fig. S1*B*) immunofluorescence staining showed similar levels of total and cell-surface expression, respectively, between hGPR17L-WT and hGPR17L variants. In addition, we also tested the nonsynonymous mutations at corresponding residues in mouse Gpr17, which is 28 amino acid shorter than the human GPR17 long isoform. We used dual-tagged mouse Gpr17 constructs (*i.e.*, N-terminal HA-epitope tagged and C-terminal GFP fusion) to quantify the cell surface and cytoplasmic receptor localization (Fig. S2*A*). Our results revealed similar cell-surface to total receptor expression ratios for mGPR17-S15L, -D77N, -R273H, and -G326V (corresponding to hGPR17L variants -F43L, -D105N, -R301H, and -G354V, respectively) as compared with mGPR17-WT (Fig. S2, *B* and *C*). Together, our results showed that these nonsynonymous GPR17 variants have similar protein expression, subcellular trafficking, and localization as compared with the wild-type GPR17.

### Human GPR17 variants had distinct cAMP signaling profiles

**Figure 3:**
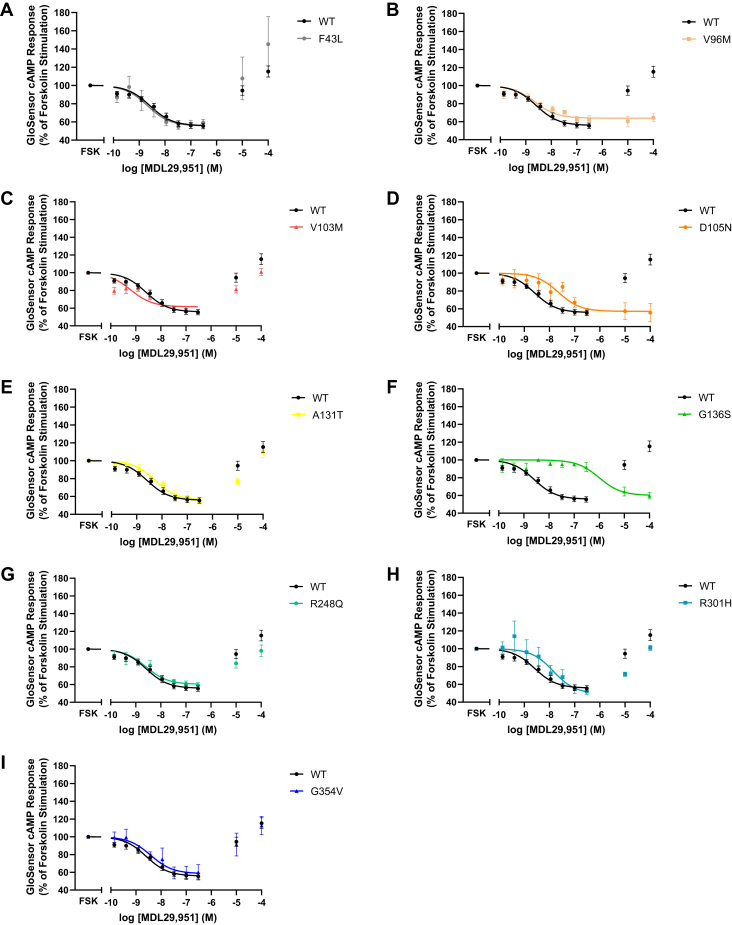
**Human GPR17 variants have distinct cAMP signaling profiles.** HEK293 cells transiently expressing hGPR17L-WT or -variants and GloSensor-cAMP-22F were treated with 3 μM forskolin and MDL29,951 (ranging from 0.15 nM to 100 μM) and the luminescent GloSensor cAMP response was measured. Data points represent mean ± SEM of three to ten independent experiments and are graphical representations of hGPR17L-WT together with individual variants hGPR17L-F43L (*A*), hGPR17L-V96M (*B*), hGPR17L-V103M (*C*), hGPR17L-D105N (*D*), hGPR17L-A131T (*E*), hGPR17L-G136S (*F*), hGPR17L-R248Q (*G*), hGPR17L-R301H (*H*), and hGPR17L-G354V (*I*). The pIC_50_ and efficacy values for corresponding experiments were reported in Table 2.

GPR17 is reported to couple to Gαi/o and Gαs to modulate cAMP signaling (19). The cAMP signaling properties of the human GPR17L variants were evaluated using a live-cell luciferase-based cAMP biosensor assay upon transient transfection of receptor in HEK293 cells. The constitutive regulation of cAMP by GPR17 expression was measured under basal conditions (Fig. S3*A*) and upon stimulation with the direct adenylyl cyclase activator, forskolin (Fig. S3*B*). No significant differences were observed in the basal or forskolin-stimulated cAMP levels in cells expressing hGPR17L variants as compared with hGPR17L-WT, suggesting similar levels of constitutive cAMP regulation (Fig. S3). To observe agonist-mediated GPR17 signaling, cells were treated with varying concentrations of the synthetic agonist, MDL29,951, and subsequently stimulated with forskolin. As expected, MDL29,951-mediated hGPR17L signaling inhibited forskolin-stimulated cAMP production in a concentration-dependent manner with a pIC_50_ value of 8.52 ± 0.13 and maximum inhibition of 47 ± 2.5% (Table 2). Consistent with previous studies in HEK293 cells (19), we also observed a bell-shaped dose–response curve with the treatment of MDL29,951. For example, although lower concentrations of MDL29,951 (*i.e.*, <300 nM) inhibited forskolin-stimulated cAMP production, higher concentrations of MDL29,951 (*i.e.*, 10 μM and 100 μM) displayed no inhibition of forskolin-stimulated cAMP production. For each GPR17 variant tested, we observed one of three distinct patterns of cAMP signaling profiles according to the shape of their dose-response curve and potency of MDL29,951. First, hGPR17L variants had similar bell-shaped dose–response curves and potency as compared with wild-type hGPR17L (Table 2, Fig. 3). Six hGPR17L variants demonstrated this pattern, including F43L (Fig. 3*A*), V103M (Fig. 3*C*), A131T (Fig. 3*E*), R248Q (Fig. 3*G*), R301H (Fig. 3*H*), and G354V (Fig. 3*I*). Second, an hGPR17L variant had a sigmoidal dose–response curve and similar potency as compared with wild-type hGPR17L. Human GPR17L-V96M had this pattern, as it displayed agonist-stimulated inhibition of cAMP that was similar to hGPR17L-WT with regard to potency and maximum inhibition, but without the loss of cAMP inhibition at higher concentrations of MDL29,951 (Table 2, Fig. 3*B*). Third, hGPR17L variants had sigmoidal dose–response curves and decreased potency as compared with wild-type hGPR17L. Two variants had this pattern, including D105N (Fig. 3*D*) and G136S (Fig. 3*F*). For example, in cells expressing hGPR17L-G136S, MDL29,951 treatment inhibited forskolin-stimulated cAMP with a pIC_50_ value of 5.89 ± 0.30, representing activity that was approximately 400-fold less potent relative to hGPR17L-WT (Table 2, Fig. 3*F*). Also, MDL29,951 treatment was sixfold less potent for inhibition of forskolin-stimulated cAMP in cells expressing hGPR17L-D105N (Table 2, Fig. 3*D*). In summary, hGPR17L-V96M, -D105N, and -G136S have distinctly altered agonist-modulated cAMP signaling profiles.

**Table 2:** Human GPR17L variant-mediated cAMP signaling

Variant	cAMP			
	IC_50_ (nM [pIC_50_ ± SEM])	Efficacy (% inhibition)	10 μM MDL +3 μM FSK (% of FSK)	100 μM MDL +3 μM FSK (% of FSK)
WT	3.0 (8.52 ± 0.13)	47 ± 2.5	92 ± 4.4	115 ± 6.1
F43L	1.6 (8.81 ± 0.37)	44 ± 2.7	107 ± 24	145 ± 30
V96M	1.7 (8.77 ± 0.36)	36 ± 4.2	60 ± 5.6	65 ± 4.6
V103M	0.57 (9.24 ± 0.41)	40 ± 1.3	81 ± 4.0	101 ± 4.0
D105N	18 (7.75 ± 0.25)	48 ± 4.7	49 ± 7.3	56 ± 10
A131T	4.9 (8.31 ± 0.13)	44 ± 4.5	77 ± 3.5	109 ± 5.3
G136S	1300 (5.89 ± 0.30)	39 ± 2.3	65 ± 5.0	60 ± 3.4
R248Q	1.9 (8.73 ± 2.2)	41 ± 3.9	84 ± 5.5	98 ± 6.7
R301H	10 (8.00 ± 0.46)	52 ± 5.2	72 ± 2.5	101 ± 2.9
G354V	4.6 (8.34 ± 0.25)	42 ± 6.7	91 ± 13	113 ± 10

HEK293 cells transiently expressing hGPR17L-WT or -variants and GloSensor-cAMP-22F were treated with 3 μM forskolin and MDL29,951 (ranging from 0.15 nM to 100 μM) and the luminescent GloSensor cAMP response was measured. Means of duplicate data points were fit to a sigmoidal dose–response equation for the inhibitory phase of the concentration–response curve (*i.e.*, for 0.1 nM–1 μM MDL29,951). Mean ± SEM of IC_50_ (nM [pIC_50_ ± SEM]), efficacy (% inhibition), and GloSensor cAMP responses for 10 μM or 100 μM MDL29,951 + 3 μM forskolin (% of forskolin response) are reported for three to ten independent experiments.

FSK, forskolin; MDL, MDL29,951.

### Human GPR17 variants had distinct stimulatory cAMP responses

**Figure 4:**
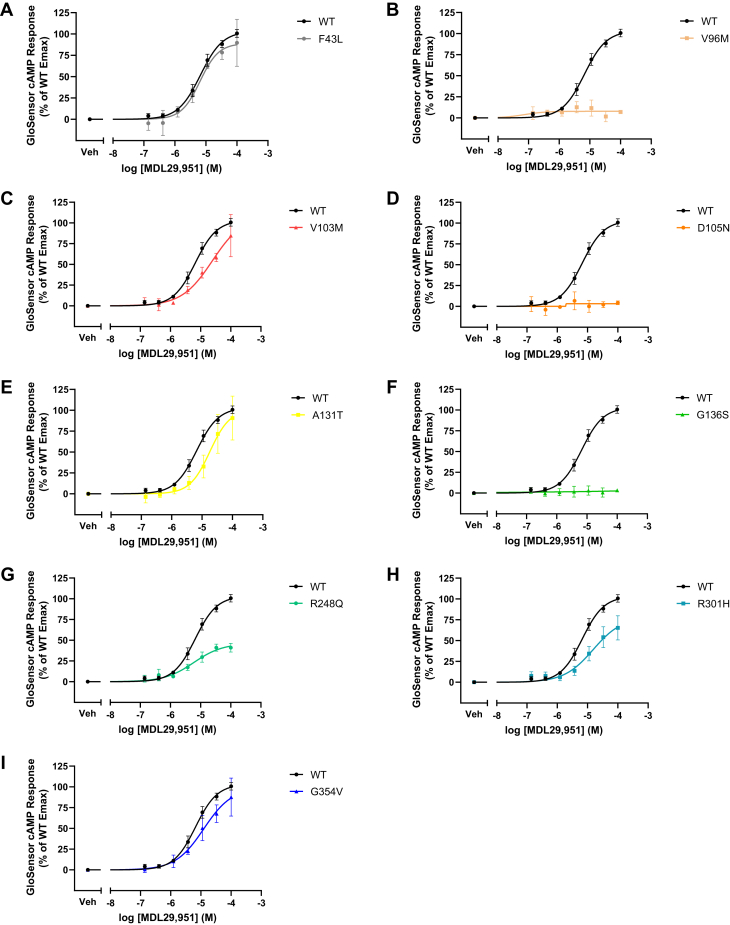
**Human GPR17 variants have distinct stimulatory cAMP responses.** HEK293 cells were transfected with hGPR17L-WT or -variants and GloSensor-cAMP-22F, pretreated with 100 ng/ml pertussis toxin overnight, and subsequently stimulated with MDL29,951 (ranging from 140 nM to 100 μM). Data points represent the mean ± SEM luminescent GloSensor cAMP responses from three independent experiments and are graphical representations of hGPR17L-WT together with individual variants hGPR17L-F43L (*A*), hGPR17L-V96M (*B*), hGPR17L-V103M (*C*), hGPR17L-D105N (*D*), hGPR17L-A131T (*E*), hGPR17L-G136S (*F*), hGPR17L-R248Q (*G*), hGPR17L-R301H (*H*), and hGPR17L-G354V (*I*). The pEC_50_ and efficacy for corresponding experiments were reported in Table 3.

The hGPR17L-mediated regulation of cAMP by higher concentrations of MDL29,951 (*e.g.*, 100 nM–100 μM) was investigated further in the absence of forskolin. In cells expressing hGPR17L-WT, MDL29,951 treatment provided both inhibitory and stimulatory cAMP responses that were dependent on the concentration of MDL29,951 (Fig. S4). Specifically, ∼100–300 nM MDL29,951 decreased cAMP levels, whereas higher concentrations of MDL29,951 were able to stimulate increases of cAMP. This cAMP response was also studied independently from the GPR17-mediated inhibition of cAMP by uncoupling Gαi/o subunits from the receptor with pertussis toxin. Cells that were pretreated with 100 ng/ml pertussis toxin overnight exclusively had a stimulatory response to MDL29,951 with a pEC_50_ of 5.18 ± 0.04 (Table 3 and Fig. S4) and maximum response of 1.21 ± 0.24 ΔRLU/RLU, consistent with pertussis toxin functioning to prevent Gαi/o coupling to hGPR17L. These data suggest that MDL29,951 promoted stimulatory cAMP responses from hGPR17L that are independent from Gαi/o-mediated signaling pathways. The hGPR17L variants were evaluated in the presence of pertussis toxin with respect to the stimulatory cAMP response to MDL29,951 treatment (Table 3 and Fig. 4). Several different patterns of the stimulatory cAMP responses were observed for hGPR17L variants. For example, hGPR17L-F43L (Fig. 4*A*), -R301H (Fig. 4*H*), and -G354V (Fig. 4*I*) had agonist-induced stimulatory cAMP responses that were similar to hGPR17L-WT. However, modest losses of potency (*i.e.*, ∼3–7-fold shift in EC_50_) were observed for MDL29,951-stimulated increases in cAMP mediated by hGPR17L-V103M (Fig. 4*C*) and -A131T (Fig. 4*E*). Also, MDL29,951 treatment was less efficacious for elevating cAMP through hGPR17L-R248Q (Fig. 4*G*). The most striking effects were observed for hGPR17L-V96M (Fig. 4*B*), -D105N (Fig. 4*D*), and -G136S (Fig. 4*F*), as MDL29,951 was unable to promote stimulatory cAMP responses mediated by these hGPR17L variants.

**Table 3:** Human GPR17L variant-mediated stimulatory cAMP signaling

Variant	Stimulatory cAMP	
	EC_50_ (μM [pEC_50_ ± SEM])	Efficacy (% of WT Emax)
WT	6.6 (5.18 ± 0.04)	100
F43L	6.2 (5.21 ± 0.13)	99 ± 20
V96M	NA	NA
V103M	47 (4.33 ± 0.36)	150 ± 48
D105N	NA	NA
A131T	19 (4.72 ± 0.05)	105 ± 21
G136S	NA	NA
R248Q	6.1 (5.22 ± 0.01)	41 ± 1.7
R301H	16 (4.84 ± 0.12)	68 ± 11
G354V	14 (4.88 ± 0.12)	105 ± 23

HEK293 cells transiently expressing hGPR17L-WT or -variants and GloSensor-cAMP-22F were pretreated with 100 ng/ml pertussis toxin overnight and subsequently stimulated with MDL29,951 (ranging from 140 nM to 100 μM) and the luminescent GloSensor cAMP response was measured. Means of duplicate data points were fit to a sigmoidal dose–response equation and mean ± SEM of EC_50_ (μM [pEC_50_ ± SEM]) and efficacy (% of WT Emax ± SEM) are reported for three independent experiments.

NA, not applicable.

### Human GPR17 variants had distinct calcium signaling activities

**Figure 5:**
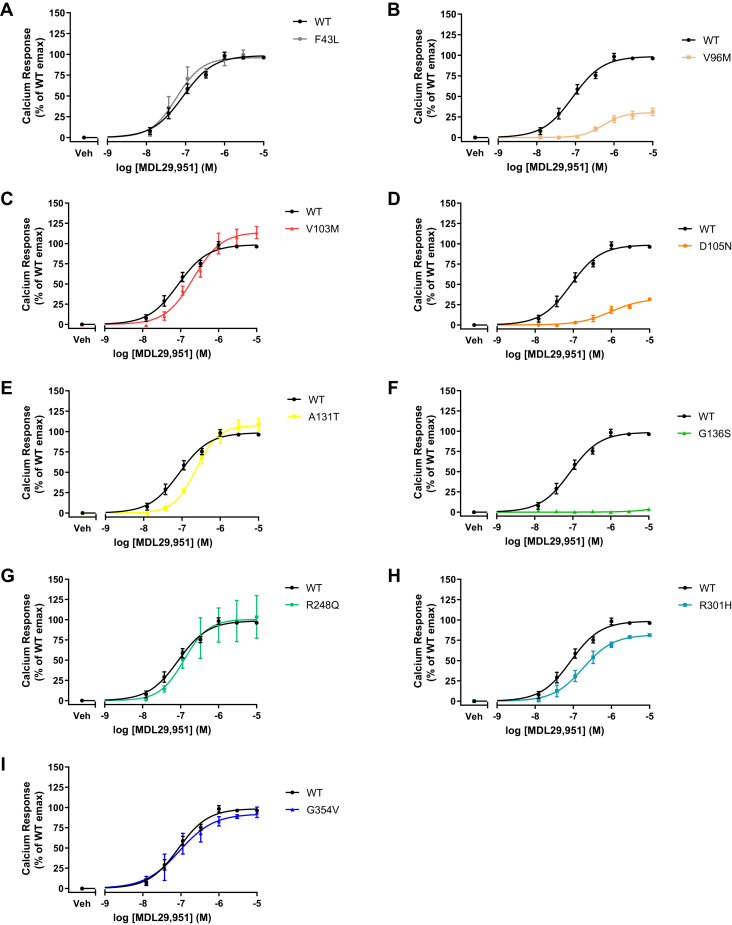
**Human GPR17 variants have distinct calcium signaling.** HEK293 cells transiently expressing hGPR17L-WT or -variants were stimulated with MDL29,951 (ranging from 4.1 nM to 10 μM) and the calcium response was measured. Data points represent mean ± SEM for three to six independent experiments, were fit to a sigmoidal dose–response equation, and are graphical representations of hGPR17L-WT together with individual variants hGPR17L-F43L (*A*), hGPR17L-V96M (*B*), hGPR17L-V103M (*C*), hGPR17L-D105N (*D*), hGPR17L-A131T (*E*), hGPR17L-G136S (*F*), hGPR17L-R248Q (*G*), hGPR17L-R301H (*H*), and hGPR17L-G354V (*I*). The pEC_50_ and efficacy values for corresponding experiments were reported in Table 4.

Human GPR17L activation is also known to promote calcium flux (19). The downstream calcium signaling of hGPR17L variants was investigated by transient transfection in HEK293 cells and measuring live-cell calcium responses. Upon expression of hGPR17L variants, no significant differences in basal calcium levels were observed as compared with hGPR17L-WT (Fig. S5). MDL29,951 treatment stimulated hGPR17L-mediated calcium mobilization in a concentration-dependent manner with a pEC_50_ value of 7.09 ± 0.11 (Table 4) and maximum stimulation of 1.51 ± 0.14 Peak ΔF/F. MDL29,951-stimulated calcium responses for hGPR17L-F43L, -V103M, -A131T, -R248Q, -R301H, and -G354V were similar to that of hGPR17L-WT (Table 4, Fig. 5, *A*, *C*, *E*, and *G–I*, respectively). In contrast, agonist-mediated calcium signaling was impaired for hGPR17L-V96M, -D105N, and -G136S. Specifically, hGPR17L-V96M and -D105N had less potent (approximately 6–7-fold) and less efficacious (approximately 70% reduction of efficacy) calcium responses to MDL29,951 treatment as compared with hGPR17L-WT (Table 4, Fig. 5, *B* and *D*). Also, we observed a near complete loss of hGPR17L-G136S-mediated calcium signaling, as 10 μM MDL29,951 provided a calcium response that was 3.6 ± 1.3% of the maximum response observed for hGPR17L-WT (Table 4, Fig. 5*F*). Our data suggest that calcium signaling was impaired for three hGPR17L variants (V96M, D105N, and G136S).

**Table 4:** Human GPR17L variant-mediated calcium signaling

Variant	Ca^2+^	
	EC_50_ (nM) (pEC_50_ ± SEM)	Efficacy (% of WT Emax)
WT	82 (7.09 ± 0.11)	100
F43L	57 (7.25 ± 0.19)	97 ± 3.9
V96M	540 (6.27 ± 0.05)	31 ± 4.7
V103M	220 (6.66 ± 0.09)	119 ± 8.4
D105N	620 (6.21 ± 0.11)	28 ± 4.5
A131T	230 (6.63 ± 0.08)	109 ± 8.9
G136S	NA	NA
R248Q	120 (6.92 ± 0.02)	103 ± 25
R301H	180 (6.74 ± 0.14)	85 ± 3.6
G354V	89 (7.05 ± 0.29)	92 ± 2.6

HEK293 cells transiently expressing hGPR17L-WT or -variants were stimulated with MDL29,951 (ranging from 4.1 nM to 10 μM) and the calcium response was measured. Means of duplicate data points were fit to a sigmoidal dose–response equation and mean ± SEM of EC_50_ (nM [pEC_50_ ± SEM]) and efficacy (% of WT Emax ± SEM) are reported for three to six independent experiments.

NA, not applicable.

It is notable that in addition to Gq-mediated calcium mobilization, several Gi/o-coupled receptors can mediate calcium flux *via* Gβγ subunits (27, 28, 29). As GPR17-stimulated calcium mobilization is mediated by both Gq and Gi/o-Gβγ subunits (28), we investigated the contributions of Gi/o-Gβγ subunits to hGPR17L-V96M and -D105N variant-mediated calcium mobilization by using pertussis toxin to inhibit Gi/o-Gβγ subunits (Fig. S6). Consistent with a previous report (28), pertussis toxin treatment had modest effects on MDL29,951-stimulated hGPR17L-WT calcium flux, with reduced efficacy (82 ± 1.4% of control pretreatment) and potency (approximately 2.5-fold shift in EC_50_). Furthermore, reduced calcium mobilization was also observed for hGPR17L-V96M and hGPR17L-D105N in the presence of pertussis toxin, suggesting that Gi/o-Gβγ subunits contribute to calcium responses for these hGPR17L variants.

### Human GPR17 variants displayed diverse β-arrestin recruitment

**Figure 6:**
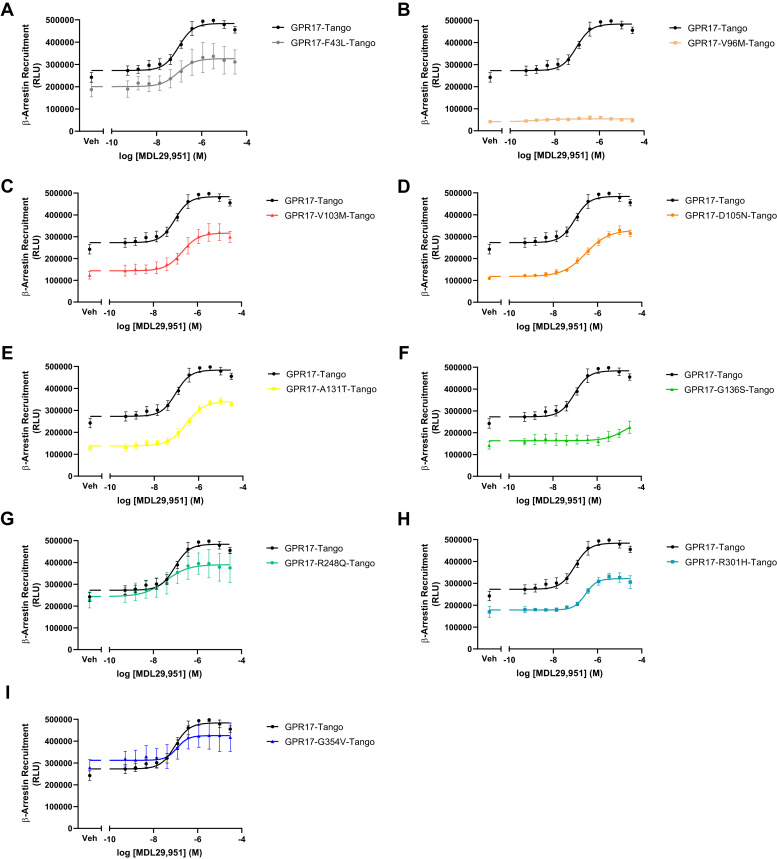
**Human GPR17 variants display diverse signaling activities through β-arrestin recruitment.** HTLA cells transiently transfected with hGPR17L-WT-Tango or hGPR17L -Tango variants constructs were stimulated with MDL29,951 (ranging from 0.5 nM to 30 μM) and the luciferase reporter response was measured. Data points represent mean±SEM for three independent experiments, were fit to a sigmoidal dose–response equation, and are graphical representations of hGPR17L-WT-Tango together with individual variants hGPR17L-F43L-Tango (*A*), hGPR17L-V96M-Tango (*B*), hGPR17L-V103M-Tango (*C*), hGPR17L-D105N-Tango (*D*), hGPR17L-A131T-Tango (*E*), hGPR17L-G136S-Tango (*F*), hGPR17L-R248Q-Tango (*G*), hGPR17L-R301H-Tango (*H*), and hGPR17L-G354V-Tango (*I*). The pEC_50_ and efficacy values for corresponding experiments were reported in Table 5.

GPR17 activation is known to stimulate β-arrestin recruitment to promote receptor internalization and mediate noncanonical β-arrestin signaling (19, 22). The ability of hGPR17L variants to recruit β-arrestin was tested using the TANGO β-arrestin recruitment assay following transient transfection of receptor-Tango constructs in HTLA cells (30, 31). Expression of hGPR17L-WT-Tango and hGPR17L-Tango variants in HTLA cells resulted in a spread of basal luminescence signals (Figure 6 and Fig. S7). Specifically, basal luminescence signals were significantly decreased for hGPR17L-V96M-, -V103M-, -D105N-, -A131T-, and -G136S-Tango, suggesting differences in constitutive β-arrestin recruitment (Fig. S7). Such differences in basal β-arrestin recruitment do not appear to reflect differences in receptor expression levels, as western blotting in HTLA cells revealed no significant differences in the expression levels of hGPR17L-Tango variants as compared with hGPR17L-WT-Tango (Fig. S8). Agonist-stimulated β-arrestin recruitment was also measured for hGPR17L-WT-Tango and hGPR17L-Tango variants. MDL29,951 treatment yielded a concentration-dependent increase in hGPR17L-WT-Tango-mediated β-arrestin recruitment with a pEC_50_ value of 7.02 ± 0.02 (Table 5) and maximum response of 1.78 ± 0.09-fold over baseline. Several hGPR17L-Tango variants, including hGPR17L-F43L- (Fig. 6*A*), -V103M- (Fig. 6*C*), -D105N- (Fig. 6*D*), -A131T- (Fig. 6*E*), -R248Q- (Fig. 6*G*), -R301H- (Fig. 6*H*), and -G354V-Tango (Fig. 6*I*), mediated MDL29,951-stimulated β-arrestin recruitment in a concentration-dependent manner similar to that observed for hGPR17L-WT-Tango. For example, the EC_50_ values for MDL29,951 stimulation of β-arrestin recruitment mediated by each of these hGPR17L-Tango variants were less than approximately threefold different than that observed for hGPR17L-WT-Tango (Table 5). Furthermore, each of these hGPR17L-Tango variants had maximum luminescent responses (Fig. S9) and concentration–response curve spans (Table 5) that were not significantly different than hGPR17L-WT-Tango, suggesting similar efficacy for β-arrestin recruitment in response to MDL29,951 treatment. In contrast, hGPR17L-V96M- (Fig. 6*B*) and -G136S-Tango (Fig. 6*F*) demonstrated impaired agonist-stimulated β-arrestin recruitment. Specifically, hGPR17L-V96M-Tango (Fig. 6*B*) had no apparent agonist-stimulated β-arrestin recruitment and hGPR17L-G136S-Tango had only a modest response to 30 μM MDL29,951 (Fig. 6*F*), while both variants had maximum luminescent responses that were significantly lower than hGPR17L-WT-Tango (Fig. S9). In summary, β-arrestin recruitment mediated by hGPR17L-V96M- and -G136S-Tango was generally impaired; however, β-arrestin recruitment mediated by several other hGPR17L-Tango variants appeared altered in a complex manner. For example, hGPR17L-V103M-, -D105N-, and -A131T-Tango had impaired basal β-arrestin recruitment (Fig. S7), but retained MDL29,951-stimulated β-arrestin recruitment at similar levels to hGPR17L-WT-Tango (Table 5 and Fig. S9).

**Table 5:** Human GPR17L variant-mediated β-arrestin recruitment

Variant	β-arrestin recruitment	
	EC_50_ (nM [pEC_50_ ± SEM])	Efficacy (% of WT span)
WT	96 (7.02 ± 0.02)	100
F43L	83 (7.08 ± 0.03)	61 ± 13
V96M	NA	NA
V103M	210 (6.69 ± 0.05)	84 ± 13
D105N	290 (6.53 ± 0.03)	100 ± 5.0
A131T	280 (6.55 ± 0.06)	97 ± 5.2
G136S	NA	NA
R248Q	28 (7.55 ± 0.16)	73 ± 16
R301H	280 (6.55 ± 0.04)	69 ± 9.0
G354V	110 (6.96 ± 0.04)	64 ± S22

HTLA cells transiently transfected with hGPR17L-WT-Tango or hGPR17L-Tango variants were stimulated with MDL29,951 (ranging from 0.5 nM to 30 μM) and the luciferase reporter response was measured. Mean values of duplicate data points were fit to a sigmoidal dose–response equation. Mean ± SEM of EC_50_ (nM [pEC_50_ ± SEM]) and efficacy (% of WT span) are reported for three independent experiments.

NA, not applicable.

### GPR17 homology model

**Figure 7:**
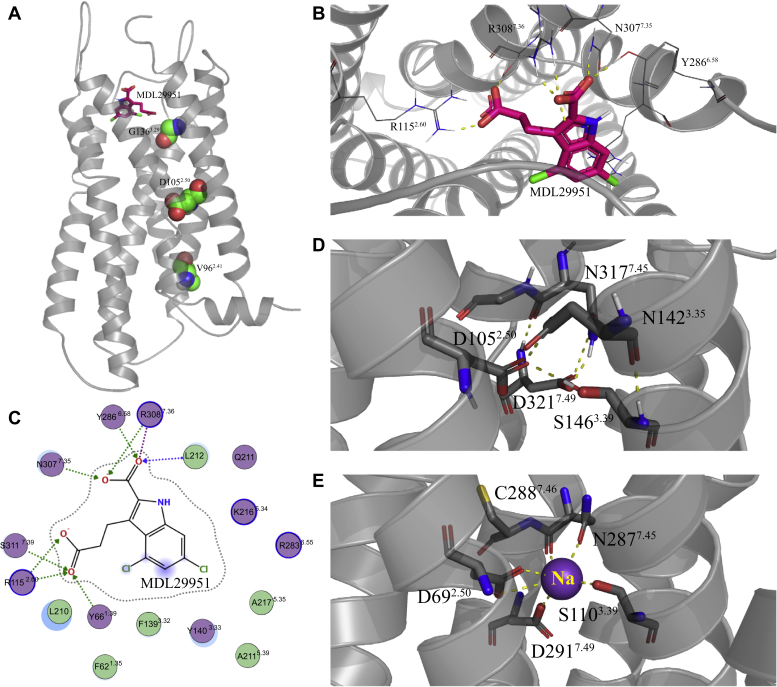
**Homology modeled structure of GPR17.***A*, Ribbon diagram representation of the modeled GPR17 structure with MDL29,951 (shown as stick structure) docked and variant amino acids V96^2.41^, D105^2.50^, and G136^3.29^ represented as spheres. *B*, proposed binding orientation of MDL29,951 docked in the putative ligand-binding site of GPR17. MDL29,951 is represented as *sticks* and the structure of GPR17 is depicted as a ribbon diagram with ligand-interacting residues shown with *stick models*. *C*, two-dimensional interaction summary of MDL29,951 with GPR17 residues in the ligand-binding pocket. *D*, hydrogen bonding network in the modeled GPR17 structure around residue D105^2.50^ in the absence of sodium ion. *E*, sodium-binding pocket in CysLT1R (PDBID: 6RZ4) with *dashed yellow lines* indicating favorable electrostatic interactions.

Several human GPR17 variants identified in individuals with metabolic diseases had altered downstream signaling. Therefore, we generated a homology model of GPR17 to evaluate the location and structural context of the variant amino acids and to propose potential molecular mechanisms for such altered functional signaling. The homology model of GPR17 was constructed using an X-ray crystal structure of human cysteinyl leukotriene receptor 2 (CysLT2R, PDBID: 6RZ6) (32) as a template (see Fig. S10 for sequence alignment) and the GPR17 agonist, MDL29,951, was docked in the ligand-binding site. Here, we also included a generic structure-based numbering system (Ballesteros–Weinstein numbering scheme) to facilitate comparison of amino acid positions across GPCRs (*e.g.*, D105 of hGPR17L corresponds to structure-based coordinate 2.50 and is denoted D105^2.50^) (33, 34). V96^2.41^, D105^2.50^, and G136^3.29^ are located in transmembrane (TM) domains and can be evaluated using the modeled GPR17 structure (Fig. 7*A*). The putative agonist-binding orientation suggested that MDL29,951 binding was stabilized largely by electrostatic and hydrogen bonding interactions with the extracellular ends of TM2, TM6, and TM7 of GPR17. Specifically, the carboxylic acid of indole position 2 of the ligand was predicted to form an electrostatic interaction with R308^7.36^ and hydrogen bonding interactions with N307^7.35^ and Y286^6.58^ (Fig. 7, *B* and *C*). Furthermore, the docking suggested an electrostatic interaction between the terminal carboxylic acid at indole position 3 and R115^2.60^ (Fig. 7, *B* and *C*). The locations of the hGPR17L variant residues were found at different depths of the model relative to the putative MDL29,951-binding pocket. For example, V96^2.41^ was located at the cytoplasmic end of TM2 and may be in contact with the cytoplasmic end of TM4. It is possible that hGPR17L-V96M^2.41^ has altered hydrophobic packing between TM2 and TM4 (Fig. 7*A*). D105^2.50^ was located near the middle of TM2 and was positioned relatively deeper within the receptor than the putative MDL29,951-binding site. Our homology model of GPR17 was built in the absence of sodium ion and suggested that D105^2.50^ forms a hydrogen bond network with residues N77^1.50^, N142^3.35^, S146^3.39^, N317^7.45^, and D321^7.49^ (Fig. 7*D*). However, D^2.50^ is a highly conserved anchor residue for coordination of sodium ions in class A GPCRs (35, 36). Four residues (D^2.50^, S^3.39^, N^7.45^, and D^7.49^) that chelate the sodium ion in CysLT1R crystal structures (37) are identical in GPR17, suggesting the possibility of sodium coordination at D105^2.50^ of GPR17 (Fig. 7*E*). Therefore, our data suggest that D105^2.50^ is engaged in stabilization of the TM domains through a hydrogen bonding network or in binding a sodium ion, and that the hGPR17L-D105N^2.50^ variant may perturb such interactions. Notably, of the variant residues, G136^3.29^ was modeled to be in closest proximity to the MDL29,951-binding site. It is possible that hGPR17L-G136S^3.29^ has impaired MDL29,951 binding due to an increase in steric bulk of the serine residue as compared with glycine (Fig. 7*A*). Taken together, our GPR17 homology model provided structural information for potential mechanisms underlying the distinctly altered signaling activities for hGPR17L-V96M^2.41^, -D105N^2.50^, and -G136S^3.29^ variants.

## Discussion

**Figure 8:**
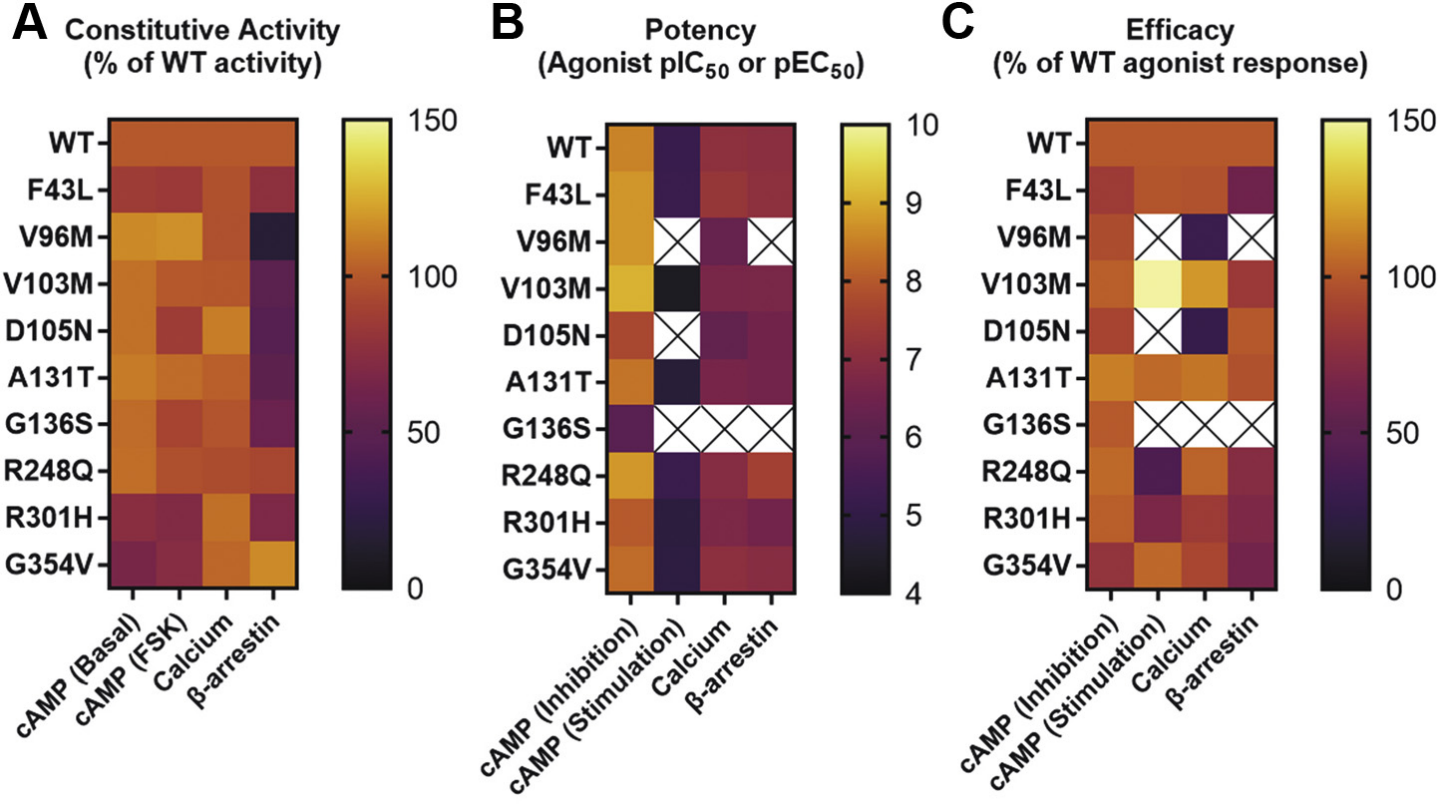
**Summary of human GPR17L variant signaling profiles.** Heat maps are graphical representations of GPR17-mediated cAMP inhibition, cAMP stimulation, calcium mobilization, and β-arrestin recruitment data reported for (*A*) mean constitutive activity expressed as a percentage of the measured signal for hGPR17L-WT, (*B*) mean MDL29,951 potency (*i.e.*, pIC_50_ or pEC_50_), and (*C*) mean MDL29,951 efficacy where data represent the difference between baseline and maximum MDL29,951-stimulated signaling responses and are reported as a percentage of the agonist-stimulated hGPR17L-WT response.

Whole-body germline knockout and cell-type specific conditional genetic ablation studies in rodents have linked GPR17 function in distinct cell types in the central nervous system (CNS) and peripheral organs to the regulation of metabolism (11, 12, 13). However, the genetic contribution of GPR17 to human metabolic regulation has yet to be reported. The present study identified naturally occurring nonsynonymous *GPR17* variants in healthy individuals as well as those with clinical metabolic deficits. Recent studies suggest the possibility that naturally occurring GPCR missense variants that have altered functional signaling profiles may be associated with altered risk of disease outcomes (25, 26). Therefore, we studied nine nonsynonymous human GPR17 variants to evaluate receptor expression levels, subcellular trafficking, and functional downstream cAMP, calcium, and β-arrestin signaling pathways. Notably, several GPR17 variants identified in individuals with metabolic deficits had altered downstream signaling profiles (Fig. 8).

A variety of possible molecular changes, or a combination thereof, may contribute to altered GPR17 variant signaling function. For example, changes in receptor expression level, receptor trafficking and subcellular localization, ligand binding, conformational changes associated with receptor activation, and receptor–transducer coupling may contribute to altered GPR17 signaling. However, evaluation of GPR17 variant expression levels and localization by western blotting and immunostaining indicated that the overall expression levels and localization were similar between the tested GPR17 variants and hGPR17L-WT, suggesting that there were no global perturbations that would lead to misfolded and degraded receptors or improperly trafficked receptors. Furthermore, agonist-stimulated signaling was similar to hGPR17L-WT for at least one signaling pathway for all variants that were evaluated with the exception of hGPR17-G136S, further supporting unperturbed receptor folding and trafficking, but also suggesting generally preserved ligand-binding properties in eight of the nine GPR17 variants evaluated.

Interestingly, for hGPR17L-G136S, both efficacy and potency were impaired for MDL29,951 modulation of all downstream signaling pathways that were tested (*i.e.*, cAMP, Ca^2+^, and β-arrestin recruitment), suggesting a global signaling impairment for this variant (Fig. 8). Furthermore, consistent with a previously published GPR17 homology model based on an active state P2RY12 crystal structure (38), our GPR17 homology model suggests that G136 resides in close proximity to the putative MDL29,951-binding pocket. It is possible that the added steric bulk associated with substitution of glycine for serine perturbs the receptor-MDL29,951 interaction. Taken together, our results suggest that the impaired signaling function observed for hGPR17L-G136S is likely due to disrupted MDL29,951 binding.

Human GPR17L-D105N demonstrated a unique pattern of signaling function (Fig. 8). Both agonist-mediated inhibition of cAMP and stimulation of Ca^2+^ mobilization were less potent for hGPR17L-D105N as compared with hGPR17L-WT. However, the efficacy for cAMP inhibition was similar to that of hGPR17L-WT, whereas the efficacy for stimulating Ca^2+^ flux was greatly reduced. The reversal of cAMP inhibition and stimulatory cAMP responses typically observed at the higher MDL29,951 concentrations (*i.e.*, > ∼1 μM) were absent for hGPR17L-D105N. Interestingly, constitutive β-arrestin recruitment was impaired, but the agonist-stimulated β-arrestin recruitment and overall β-arrestin recruitment levels were not significantly different for the hGPR17L-D105N variant relative to hGPR17L-WT. Our data suggest that effects of D105N on efficacy and potency are signaling pathway dependent and are unlikely due to perturbation of MDL29,951 ligand binding. In agreement with this hypothesis, D105^2.50^ is located near the middle of TM2, away from the putative MDL29,951-binding site. It is notable that D^2.50^ is a critical sodium ion-coordinating residue in a conserved allosteric sodium pocket for class A GPCRs (35, 36). Furthermore, sodium ion coordination at D105^2.50^ is supported by observations from crystal structures of other class A δ-branch GPCRs including protease-activated receptor 1 (39), protease-activated receptor 2 (40), and CysLT1R (37), where sodium coordination is mediated by two acidic residues that are also present in GPR17 (*i.e.*, D105^2.50^ and D321^7.49^). The D^2.50^ residue is proposed to be one component of a common activation mechanism for Class A GPCRs (41). An integral role of D^2.50^ in agonist-stimulated GPCR signaling is supported by literature mining that revealed impaired signaling function upon mutation of this conserved residue (35, 41). It is particularly notable that D^2.50^N is known to result in signaling bias of at least one other GPCR, the angiotensin II type I receptor (42, 43). Also, mutation of sodium ion-coordinating residues, including D^2.50^, has led to efficacy switch of δ opioid receptor antagonists into β-arrestin-biased agonists (44). Taken together with our results, these studies suggest that hGPR17L-D105N may alter the allosteric sodium ion coordination and/or disrupt the conformational changes associated with the GPR17 activation mechanism.

Human GPR17L-V96M also showed a distinct pharmacological signaling profile (Fig. 8). For example, MDL29,951 inhibited forskolin-stimulated cAMP through hGPR17L-V96M similarly to hGPR17L-WT. However, the reversal of cAMP inhibition and stimulatory cAMP responses that are typically observed at MDL29,951 concentrations greater than 1 μM were absent for hGPR17L-V96M. Furthermore, both potency and efficacy of MDL29,951 for stimulation of Ca^2+^ flux were impaired for hGPR17L-V96M. MDL29,951-stimulated β-arrestin recruitment was also reduced for hGPR17L-V96M. As V96 is located at the cytoplasmic end of TM2 and in close proximity to TM4, it is unlikely that MDL29,951 binding is altered. Rather, it is possible that GPR17-transducer coupling is selectively altered. For example, G proteins (45, 46), G-protein-coupled receptor kinases (GRK) (47), and β-arrestin (48) all engage the receptor core through extensive contacts with the cytoplasmic ends of TM helices and intracellular loops (IL). Perhaps the conformation of transducer contact residues within the cytoplasmic ends of TM2 and TM4, as well as the neighboring regions of IL1 or IL2, was modified for hGPR17L-V96M, leading to selectively altered signaling function and β-arrestin recruitment.

Collectively, hGPR17L variants displayed normal receptor expression and trafficking properties, but altered functional signaling profiles. The distinct patterns of hGPR17L-mediated effects on modulation of cAMP, Ca^2+^, and β-arrestin recruitment together with GPR17 homology modeling suggest variant-specific underlying molecular mechanisms for altered signaling function that warrant further investigation. Nonetheless, mouse GPR17 ablation studies, together with evidence for cell-type-specific second messenger signaling pathways contributing to metabolic regulation, suggest that the altered signaling imparted by GPR17 variants is predicted to impact metabolically related physiological functions including feeding behavior, energy expenditure, and glucose homeostasis.

Mouse GPR17 knockout phenotyping studies including whole-body germline knockout models (13) and conditional tissue-specific genetic ablation in AgRP neurons (11), POMC neurons (12), and oligodendrocytes (13) generally implicate GPR17 in the regulation of metabolic control. It is notable that GPR17 knockout leads to favorable metabolic phenotypes such as reduced food intake, increased sensitivity to circulating hormones, improved glucose homeostasis, and protection from high-fat-diet-induced weight gain and metabolic deficits (11, 12, 13). Therefore, we initially hypothesized that GPR17 variants that contribute to metabolic derangements would display some form of a gain of signaling function that may include enhanced potency or efficacy in response to agonist stimulation or an enhancement of constitutive activity. However, rather than gain of function, we exclusively observed loss of function with respect to GPR17 variant modulation of one or more signaling pathways. It is interesting that several cell types where GPR17 is implicated in metabolic regulation are known to be oppositely regulated by Gαi/o- and Gαq-mediated signaling. Such an observation presents an alternative hypothesis where GPR17 variants contribute to metabolic derangements by altering the precise balance of downstream signaling functions. However, it is difficult to draw firm conclusions about the link between GPR17 signaling pathways and metabolic control based on the observed functional signaling profiles of the human GPR17 variants. For example, only a subset of the GPR17 variants that were identified were evaluated. More comprehensive evaluation of GPR17 variants including those identified from individuals in the control cohorts and in both the control and metabolic disease cohorts is expected to add to our understanding of the link between GPR17 signaling function and metabolic diseases. Also, the pharmacological data for the GPR17 variants was generated by measuring GPR17 signaling modulation in response to the synthetic GPR17 agonist, MDL29,951. While an initial deorphanization study reported cysteinyl leukotrienes and uracil nucleotides as endogenous agonists for GPR17 (18), this work was subsequently disputed (19, 20), suggesting that the endogenous ligands remain unknown. Therefore, it cannot be ruled out that MDL29,951 and the endogenous agonist have different pharmacological profiles. Furthermore, GPR17 couples to multiple transducers including several Gα subunits and β-arrestin and modulates distinct and overlapping downstream signaling pathways. The precise balance of GPR17 signaling within a given cell type and how the pathways integrate to contribute to metabolic control are not well understood. However, the hGPR17L variants that display signaling bias (*i.e.*, hGPR17L-V96M and hGPR17L-D105N) are expected to be useful molecular tools for untangling the contribution of GPR17-modulated signaling pathways to physiological functions, which could have broad implications for linking GPCR signaling bias with physiological outcome.

One aim of the present study was to combine human genetic approaches with *in vitro* signaling characterization to gain insight into the potential roles of GPR17 in human metabolism. Sequence analysis for the *GPR17* coding sequences of individuals from control and metabolic disease cohorts identified 18 nonsynonymous *GPR17* variants. Whereas a subset of the *GPR17* variants were exclusively observed in control cohorts (R4W, G91R, D156Y, R344M, and S362G), most variants were detected in both control and metabolic disease cohorts (F43L, V109M, R133C, R263H, V285I), or exclusively in the metabolic disease cohorts (V96M, V103M, D105N, A131T, G136S, R248Q, R301H, G354V). Several confounding factors limit us from drawing a direct link between GPR17 signaling pathways and metabolic control based on the observed functional signaling profiles of the human GPR17 variants. First, the variants were present in the general population at a frequency of <0.5% as indicated by samples in GnomAD. The low frequency observed for each variant in the experimental sample set and the general population suggest that the identified variants are rare and therefore likely lack the necessary statistical power for analyses of genetic association with traits and/or disease states. Genetic association of *GPR17* variants with metabolic deficits is further complicated by the multifactorial etiology of metabolic diseases including type 2 diabetes, cardiovascular diseases, and obesity (24). For example, predisposition to such diseases may be influenced genetically by hundreds of loci in addition to environmental factors (24). Secondly, all variants were identified from heterozygous individuals. It is notable that genes encoding cell-surface proteins and GPCR drug targets are enriched for monoallelic expression (26, 49). Genes with monoallelic expression are epigenetically regulated at the single-cell level to exhibit mitotically stable, clone-specific relative expression of the two parental alleles (49, 50). Therefore, if *GPR17* expression is monoallelic, the relative expression of wild-type and variant alleles may be heterogeneous between individuals with the same variant and between different tissues of the same individual, suggesting that a wild-type allele may not always compensate for a variant allele with altered function. Collectively, further investigation will be necessary to fully evaluate the contribution of the rare *GPR17* variant alleles to the clinical phenotypes that accompany metabolic diseases. Studies that utilize a larger sample size and investigate the allelic expression profile and balance of variant and wild-type allele expression in a given individual and tissue type are expected to provide more insight into the link between *GPR17* variants and metabolic diseases in humans. Furthermore, larger-scale human genetic approaches paired with *in vitro* signaling characterization are expected to enable grouping of rare variants that display similar functional signaling profiles providing enhanced resolution and statistical power for analyses of genetic association with clinical metabolic deficits (25).

In summary, we have identified nonsynonymous human *GPR17* genetic variants in healthy individuals and those with adverse clinical metabolic phenotypes. *In vitro* molecular characterization revealed several GPR17 variants including hGPR17L-V96M, -D105N, and -G136S that had unperturbed receptor expression and trafficking properties, but distinctly altered functional signaling profiles. GPR17 variants that display biased signaling function could represent novel molecular tools to probe the precise GPR17-mediated signaling pathways that contribute to metabolic control. Finally, the combination of human genetic approaches and *in vitro* signaling characterization is expected to contribute to our understanding of GPR17 function in human metabolism.

## Experimental procedures

### Subjects

We selected several cohorts from the UK10K project (https://www.uk10k.org/), including control cohorts and disease cohorts. Disease cohorts were selected for obesity, severe insulin resistance, and hypercholesterolemia phenotypes based on published reports of GPR17 contributing to the regulation of metabolism in mouse models. The control cohorts include 2432 samples from the Avon Longitudinal Study of Parents and Children (ALSPAC, 740 samples) and The Department of Twin Research and Genetic Epidemiology twin registry (TWINS, 1692 samples). The metabolic disease cohorts include 1622 samples and are from patients diagnosed with obesity (1372 samples), hypercholesterolemia (125 samples), and severe insulin resistance (SIR, 125 samples). Obese individuals were from two subcohorts: one of which is the Severe Childhood Onset Obesity Project (SCOOP) where individuals had BMI standard obesity scores >3 and obesity onset before the age of 10 years, with known monogenic causes excluded. The other obesity subcohort is from the Generation Scotland (GS) obesity cohort (411 samples) and contains families with extreme obese subjects, extreme obese subjects with nonobese parents, and multiple obese subjects within the same family. The Familial Hypercholesterolemia (Hyperchol) cohort has patients with consistently high–low-density lipoprotein (LDL) levels, but do not carry *APOB* or *PCSK9* mutations and have no detectable LDL receptor mutations (testing 18 common mutations and screening by single-strand conformational polymorphism and high-resolution melting for deletions/insertions).

### Sequence analysis

All variants from the *GPR17* gene were extracted from variant call formats of the relevant cohorts and mapped to SnpEffv4.0b annotations (51), and all nonsynonymous coding variants were mapped to dbNSFPv3.0 for functional prediction and annotation (52). Table 1 represents the frequency of the variants within each cohort. Most variants were too rare to perform association analyses, but we were able to compute the odds ratio for the F43L variant for two populations: (1) we compared the frequencies of the variants between the control cohorts and the diseases cohorts and (2) between the nonobese controls with all obese individuals, including the obese individuals within the control cohorts (ALSPAC and TWINS). Carrier frequencies of *GPR17* missense variants in the general population were assessed in the Genome Aggregation Database (gnomAD).

### Plasmids encoding GPR17 variants

Plasmids encoding N-terminal cleavable signal sequence (53) and HA-epitope tagged human GPR17 variants (pcDNA3-HA-hGPR17L-F43L, -D105N, -R301H, and -G354V) were constructed by site-directed mutagenesis. Briefly, the pcDNA3-HA-hGPR17 template was mutated using custom primers (Table S1) and the Q5 site-directed mutagenesis kit (E0554S; New England Biolabs). DNA containing the open reading frame was subcloned into vector backbone that was not subjected to PCR by restriction enzyme digestion at EcoRI and XhoI sites followed by ligation. Sequences were confirmed by Sanger sequencing at GenScript. Additional variants (pcDNA3-HA-hGPR17L-V96M, -V103M, -A131T, -G136S, and-R248Q) were constructed using GenScript site-directed mutagenesis services and sequences were confirmed following plasmid preparation. Furthermore, hGPR17L-variant-Tango constructs were generated for β-arrestin recruitment assays using GenScript site-directed mutagenesis services and sequences were confirmed following plasmid preparation. The GPR17-Tango template was a gift from Dr Bryan Roth (Addgene plasmid # 66336; http://n2t.net/addgene:66336; RRID:Addgene_66336).

### Cell culture and transfection

HEK293 (ATCC CRL-1573) cells were purchased from ATCC, cultured in Minimum Essential Media (MEM) supplemented with 10% fetal bovine serum and 1× Penicillin-Streptomycin solution, and maintained at 37 °C and 5% CO_2_ in a humidified incubator. HTLA cells are an HEK293T cell-derived stable cell line expressing human β-arrestin2-TEV protease fusion and tTA-dependent firefly luciferase reporter gene (30). HTLA cells were obtained from Dr Richard Axel (Columbia University), grown in DMEM, 10% fetal bovine serum, 1× Penicillin-Streptomycin solution, 100 μg/ml Hygromycin, and 2 μg/ml Puromycin, and maintained at 37 °C and 5% CO_2_ in a humidified incubator. All transfections were carried out using lipofectamine 3000 (L3000-008; Invitrogen by Thermo Fisher Scientific) according to the manufacturer's protocol.

### Western blot

HEK293 cells were seeded into wells of a 6-well plate and transfected the following day with pcDNA3.1- empty vector, pcDNA3-HA-hGPR17L, or pcDNA3-HA-hGPR17L variants using lipofectamine 3000 according to the manufacturer's protocol. Twenty-four hours posttransfection, cells were lysed with L-RIPA buffer (50 mM Tris-HCl, 150 mM NaCl, 2 mM EGTA, pH 7.5) containing 1% Triton X-100 on ice. Cells were scraped from the bottom of the plate, triturated, and sonicated. Lysate was centrifuged at 10,000*g* for 10 min to pellet cell membranes. Protein concentration of the supernatant was measured using the Pierce BCA assay (23227; Thermo Fisher Scientific), and matched protein amounts between 10 μg and 30 μg were diluted in sample buffer and separated by SDS-PAGE. Proteins were transferred to an Immobilon-FL PVDF membrane, pore size 0.45 μm (IPFL85R; MilliporeSigma). The membranes were blocked with Odyssey blocking buffer (927-50000; Li-COR) followed by incubation in primary antibodies (mouse α-HA BioLegend 16B12, 1:1000 and rabbit α-GAPDH Cell Signaling Technologies 14C10, 1:1000) overnight at 4 °C with gentle rocking. Membranes were washed with tris-buffered saline containing 0.1% tween-20 (TBS-T) three times for 5 min each and then incubated with secondary antibody for 2 h at room temperature with gentle rocking (goat α-rabbit IRDye 680RD, 1:10,000 and goat α-mouse IRDye 800CW, 1:10,000). Following three washes for 5 min each with TBS-T, membranes were allowed to dry and then imaged using a Li-COR Odyssey CLx scanner (Li-COR). Western blots were quantified using Image Studio Lite version 5.2 software (Li-COR).

Western blots for hGPR17L-WT-Tango and hGPR17L-Tango variants were conducted on lysates from transiently transfected HTLA cells and were carried out generally as specified above, with the following modifications. Matched protein amounts of 15 μg of lysates were separated by SDS-PAGE. Primary antibody (mouse α-FLAG-M2 Sigma-Aldrich F1804, 1:1000 and rabbit α-GAPDH Cell Signaling Technologies 14C10, 1:1000) incubations were for 30 min at room temperature with gentle rocking. Secondary antibody (goat α-rabbit IRDye 680RD, 1:20,000 and goat α-mouse IRDye 800CW, 1:20,000) incubations were for 1 h at room temperature with gentle rocking.

### Immunocytochemistry

HEK293 cells were seeded at 105,000 cells/chamber into poly-D-lysine treated 4-chamber microscopy slides and transfected with pcDNA3.1- empty vector, pcDNA3-HA-hGPR17L, or pcDNA3-HA-hGPR17L variants using lipofectamine 3000. After 24 h, cells were fixed with 4% paraformaldehyde and treated with either phosphate-buffered saline (PBS) or 0.1% Triton X-100 in PBS (PBST) to permeabilize the cells. Fixed cells were incubated for 30 min in Nacalai Tesque Blocking One Histo reagent at room temperature. Cells were incubated with primary antibody (1:1000 α-HA mouse antibody, Biolegend, diluted in 1:20 Blocking One Histo: PBS solution) overnight at 4 °C. Cells were washed three times with PBS for 5 min each and incubated with secondary antibody (1:500 Alexa555 goat α-mouse antibody diluted in 1:20 Blocking One Histo: PBS solution) for 1 h at room temperature. After three additional PBS washes of 5 min each, cells were counterstained with DAPI, chambers were removed, and a coverslip was mounted with VectaShield anti-fade mounting media (H-1000, Vector Laboratories) and sealed with nail polish. Slides were imaged for DAPI and Alexa555 signal using a Zeiss LSM 700 confocal microscope (Carl Zeiss). Immunocytochemistry experiments with dual N-terminal HA-epitope- and C-terminal GFP fusion-tagged GPCRs have been used to quantitatively measure the fraction of receptor in the plasma membrane of cells (54). Similarly, HA-mGpr17-GFP constructs were used to quantify the cell surface and cytoplasmic receptor localization using immunostaining as described above and imaging signals in the Alexa555, DAPI, and GFP channels using a Zeiss LSM 700 confocal microscope (Carl Zeiss).

### cAMP assay

#### Promega GloSensor cAMP assay

HEK293 cells were seeded at 20,000 cells/well into white, opaque 96-well plates. The next day, cells were transiently cotransfected with pGloSensor-cAMP-22F and pcDNA3-HA-hGPR17L or pcDNA3-HA-hGPR17L-variants using lipofectamine 3000. Twenty-four hours posttransfection, growth medium and transfection mix were aspirated and replaced with 100 μl/well equilibration medium (2% Promega GloSensor cAMP reagent [E1291; Promega Corporation] in CO_2_-independent medium) and incubated at room temperature for 2 h. Baseline luminescence was read on a Molecular Devices SpectraMax iD5 plate reader (Molecular Devices) at ambient room temperature for 8 min. Subsequently, 10 μl/well MDL29,951 diluted in assay buffer (HBSS, 20 mM HEPES, pH 7.2) was added and luminescence was read for 10 min. Finally, 10 μl/well forskolin (1099; Tocris Bioscience) diluted in assay buffer was added and luminescence was measured for 20–30 min. The average luminescence value for the 14–20 min time points for each well was normalized to their average baseline luminescence value and expressed as a percentage of the forskolin-stimulated response within each transfection condition.

For agonist-mediated stimulatory cAMP assays, HEK293 cells were seeded and transfected as noted above. Cells were treated with 100 ng/ml pertussis toxin overnight (Sigma-Aldrich P2980). Twenty-four hours posttransfection, growth medium and transfection mix were aspirated and replaced with 100 μl/well equilibration medium (2% Promega GloSensor cAMP reagent [E1291; Promega Corporation] in CO_2_-independent medium) containing 100 ng/ml pertussis toxin and incubated at room temperature for 2 h. Baseline luminescence was read every 2 min on a Molecular Devices SpectraMax iD5 plate reader (Molecular Devices) at ambient room temperature for 8 min. Subsequently, 10 μl/well MDL29,951 diluted in assay buffer (HBSS, 20 mM HEPES, pH 7.2) was added and luminescence was read every 2 min for 16 min. Within each well, the luminescence values for each time point were normalized to their average baseline luminescence values. The average luminescence values for the time points between 8 and 16 min after MDL29,951 addition were expressed as a percentage of the maximum response observed for hGPR17L-WT.

### Calcium flux assay

HEK293 cells were seeded at 20,000 cells/well into black, optical bottom, poly-D-lysine coated 96-well plates. The following day, cells were transiently transfected with pcDNA3-HA-hGPR17L or pcDNA3-HA-hGPR17L-variants using lipofectamine 3000. Twenty-four hours after transfection, the growth medium and transfection mix were decanted and 100 μl/well assay buffer (HBSS, 20 mM HEPES, pH 7.2) and 100 μl/well Calcium 6 loading dye (R8190; Molecular Devices) were added to the plate and incubated at 37 °C, 5% CO_2_ for 1 h 45 min. Plate was allowed to equilibrate to room temperature on the bench top for 30 min. MDL29,951 diluted in assay buffer was added to the wells and fluorescence (485 nm excitation, 525 nm emission, and 515 nm automatic emission cutoff) was measured using a Molecular Devices FlexStation3 (Molecular Devices). For experiments with pertussis toxin, cells were pretreated with 100 ng/ml pertussis toxin overnight and 100 ng/ml pertussis toxin was included with Calcium 6 loading dye for calcium flux assays.

### Tango β-arrestin recruitment assay

HTLA cells were seeded at 10,000 cells/well into white, opaque, poly-D-lysine-coated 96-well plates and transfected with hGPR17L-WT-Tango or hGPR17L-variant-Tango plasmids the following day using lipofectamine 3000 according to the manufacturer's protocol. The next day, cells were incubated at 37 °C and 5% CO_2_ in serum-free growth media for a 4 h serum starvation. MDL29,951 was then diluted in assay buffer (HBSS, 20 mM HEPES, pH 7.2), added to the cells, and incubated for 16 h at 37 °C and 5% CO_2_. Drug treatment was decanted and 80 μl/well of Promega Bright-Glo reagent (diluted 20× in assay buffer) (E2610; Promega Corporation) was added and incubated at room temperature for 15 min. Luminescence (1 s per well integration time) was read on a Molecular Devices SpectraMax iD5 plate reader.

### GPR17 homology model

A homology model for GPR17 was generated using the X-ray crystal structure of human cysteinyl leukotriene receptor 2 (CysLT2R, PDBID: 6RZ6) as a template. Sequence alignment, model construction, and structure refinement were carried out using the Prime module from Schrödinger (https://www.schrodinger.com/prime). The aligned sequences were manually examined to prevent gaps that would distort the helical structures in the seven transmembrane domains. The GPR17 agonist, MDL29,951, was docked to the ligand-binding site of the predicted GPR17 structure, using the Dock module in the Molecular Operation Environment (MOE) by Chemical Computing Group (http://www.chemcomp.com). The induced fit algorithm was adopted as a refinement method to allow limited flexibility for the side chains of the receptor near the ligand-binding site.

## Data availability

All data are contained within the article and references were provided for the software used in the study.

## Supporting information

This article contains supporting information.

## Conflict of interest

The authors declare that they have no conflicts of interest with the contents of this article.
