# Supplementary material for: Human GPR17 missense variants identified in metabolic disease patients have distinct downstream signaling profiles
Source: J Biol Chem. 2021 Jun 16;297(1):100881. doi: 10.1016/j.jbc.2021.100881 (PMC8267566; doi:10.1016/j.jbc.2021.100881)
Supplement: Figures S1–S10 and Table S1 [file mmc1.pdf]

Human *GPR17* missense variants identified in metabolic disease patients have unaltered cellular expression yet distinct downstream signaling profiles

Jason M. Conley[1,2], Hongmao Sun[3], Kristin L. Ayers[4,5], Hu Zhu[3], Rong Chen[4,5], Min Shen[3], Matthew D. Hall[3], and Hongxia Ren[1,2,6,7,8,9]*

[1]Herman B. Wells Center for Pediatric Research, Department of Pediatrics, Indiana University School of Medicine.
[2]Center for Diabetes and Metabolic Diseases, Indiana University School of Medicine.
[3]National Center for Advancing Translational Sciences, National Institutes of Health, Rockville, MD, 20850, USA.
[4]Department of Genetics and Genomic Sciences, The Icahn Institute for Genomics and Multiscale Biology, Icahn School of Medicine at Mount Sinai, One Gustave L. Levy Place, New York, NY, 10029, USA.
[5]Sema4, a Mount Sinai venture, Stamford, CT, 06902, USA.
[6]Department of Pharmacology & Toxicology, Indiana University School of Medicine.
[7]Stark Neurosciences Research Institute, Indiana University School of Medicine.
[8]Department of Biochemistry & Molecular Biology, Indiana University School of Medicine.
[9]Department of Anatomy, Cell Biology & Physiology, Indiana University School of Medicine.

*Corresponding author: Hongxia Ren
E-mail: renh@iu.edu
Phone: 317-274-1567
Postal address: 635 Barnhill Dr., MS2031, Indianapolis, IN 46202, USA

Running Title: GPR17 genetic variants distinctly alter signaling profiles

Keywords: metabolism, human genetics, G protein-coupled receptor (GPCR), signal transduction, metabolic disease, gene regulation, signaling, cyclic AMP (cAMP), calcium, arrestin

List of Material Included

Table S1
Figure S1
Figure S2
Figure S3
Figure S4
Figure S5
Figure S6
Figure S7
Figure S8
Figure S9
Figure S10

Table S1. Oligonucleotide sequences for site-directed mutagenesis.

Primer	Oligonucleotide Sequence (5’ to 3’)
F43L Forward	GATCACCAACCTCTCCCTGGCCAC
F43L Reverse	AGACCTGGGGGAGCCACT
D105N Forward	GGCCGTGGCCAACTTGTCGTGCG
D105N Reverse	AGATGCATCAGGAACACGTTGGCC
R301H Forward	CGCCACCCAGCACATCCTGGCCC
R301H Reverse	CAGGAGGCCCCATGGCTG
G354V Forward	CAGCTTCGAAGTGAAAACCAACGAGAGCTCGCTG
G354V Reverse	GGGGGCGGGCCCTTGAGC

Figure S1

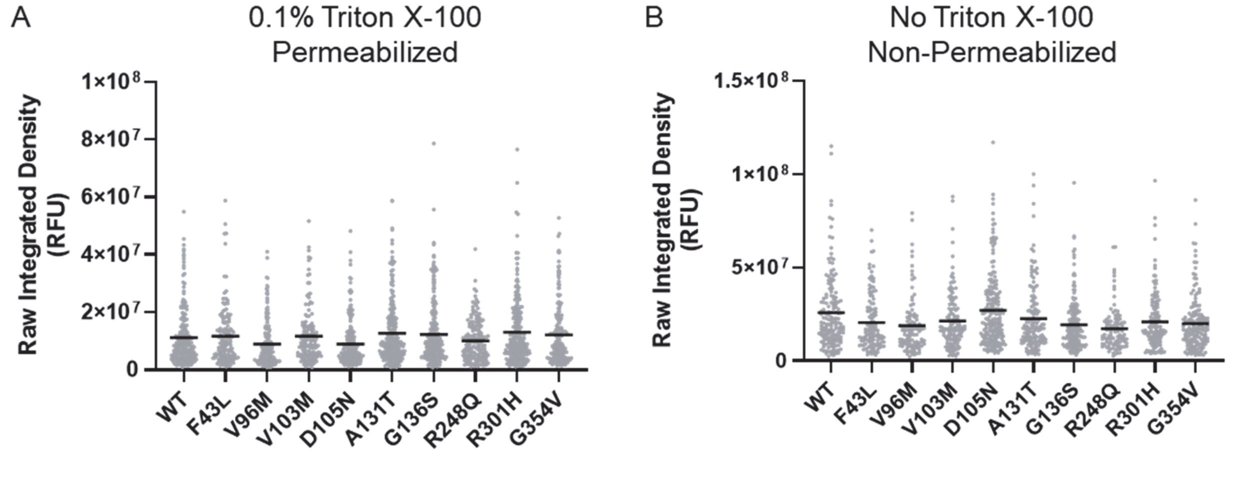

Figure S1. GPR17 variant localization. Quantification of **(A)** 0.1% Triton X-100 permeabilized total and **(B)** non-permeabilized cell-surface immunofluorescence staining of hGPR17L-WT and hGPR17L variants. Images were collected through a 20x air objective lens and imaging settings were matched for hGPR17L-WT and hGPR17L variants within each permeabilization condition. Data represent raw integrated densities for individual regions of interest that encompass approximately one cell for each data point.

Figure S2

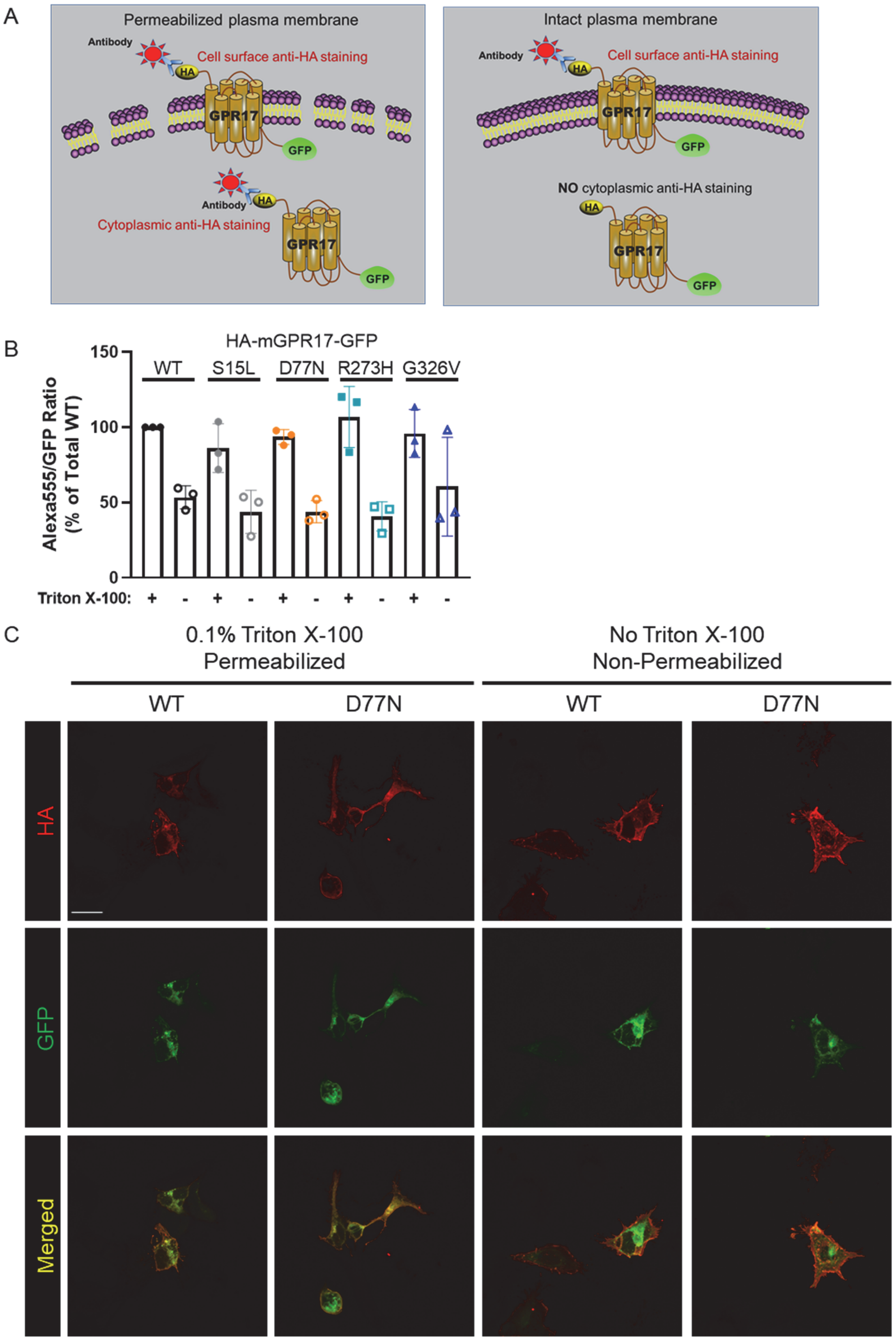
A
Permeabilized plasma membrane
Antibody
Cell surface anti-HA staining
HA
GPR17
GFP
Antibody
HA
Cytoplasmic anti-HA staining
GPR17
GFP
Intact plasma membrane
Antibody
Cell surface anti-HA staining
HA
GPR17
GFP
NO cytoplasmic anti-HA staining
HA
GPR17
GFP
B
HA-mGPR17-GFP
WT
S15L
D77N
R273H
G326V
Alexa555/GFP Ratio (% of Total WT)
150
100
50
0
Triton X-100: + - + - + - + - + -
C
0.1% Triton X-100
Permeabilized
No Triton X-100
Non-Permeabilized
WT
D77N
WT
D77N
HA
GFP
Merged

Figure S2. Dual-tagged mouse GPR17 immunofluorescence staining. (A) Schematic of dual-tagged mouse GPR17 (HA-mGPR17-GFP) immunofluorescence staining for 0.1% Triton X-100 permeabilized and non-permeabilized cells. **(B)** Quantification and **(C)** representative images of dual-tagged HA-mGPR17-GFP immunofluorescence staining of HEK293 cells. Data represent the mean±SD of Alexa555/GFP signal ratio for regions of interest from images collected through a 40x water immersion objective from three independent transfections. Scale bar, 25 µm. Statistical analysis of data in panel **(C)** by two-way ANOVA revealed significant differences between the 0.1% Triton X-100 permeabilized and non-permeabilized conditions ($p < 0.0001$), but no significant differences between variants ($p = 0.5319$) or the interaction of permeabilization condition and variants ($p = 0.3552$). Tukey's multiple comparison test revealed no significant differences between variants of the same permeabilization condition.

Figure S3

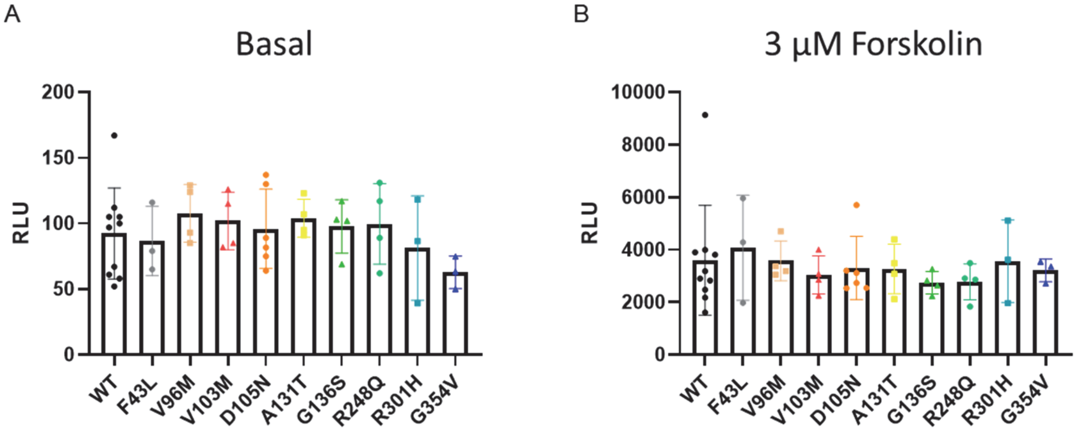

Figure S3. Human GPR17L variant expression had no effect on constitutive cAMP regulation. (A) Basal and **(B)** 3 µM forskolin-stimulated luminescent GloSensor cAMP responses were measured in HEK293 cells transiently expressing hGPR17L-WT or -variants and GloSensor-cAMP-22F. Data points represent mean±SD of three to ten independent experiments that correspond to data reported in Figure 3 and Table 2. Data were analyzed by one-way ANOVA with Dunnett's post hoc test compared to hGPR17L-WT.

Figure S4

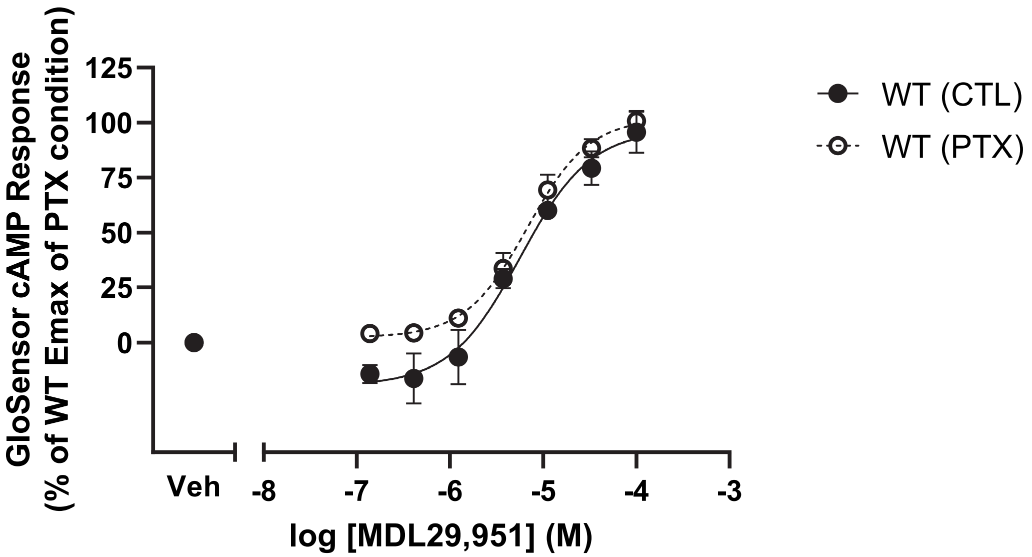

Figure S4. MDL29,951 stimulates hGPR17L-mediated cAMP production independent of Gαi/o. HEK293 cells were transfected with hGPR17L-WT and GloSensor-cAMP-22F, pretreated with vehicle or 100 ng/mL pertussis toxin overnight, and subsequently stimulated with MDL29,951. The luminescent GloSensor cAMP responses were measured. Data points were expressed as a percentage of the maximum MDL29,951 response observed for hGPR17L-WT of the pertussis toxin pretreatment condition and represent mean±SEM of three independent experiments. The hGPR17L-WT (PTX) condition data correspond to data presented in Table 3 and Figure 4. CTL, vehicle control. PTX, pertussis toxin.

Figure S5

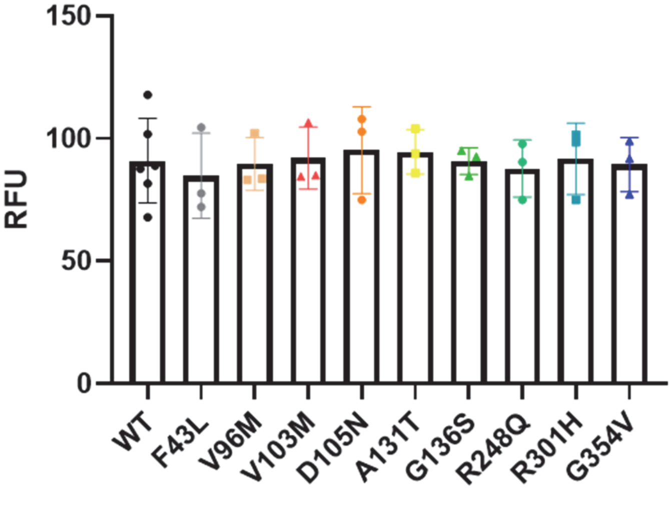

Figure S5. Human GPR17L variant expression had no effect on constitutive calcium signaling. Basal calcium signal was measured in HEK293 cells transiently expressing hGPR17L-WT or -variants. Data points represent mean±SD of three to six independent experiments that correspond to data reported in Figure 5 and Table 4. Data were analyzed by one-way ANOVA with Dunnett's post hoc test compared to hGPR17L-WT.

Figure S6

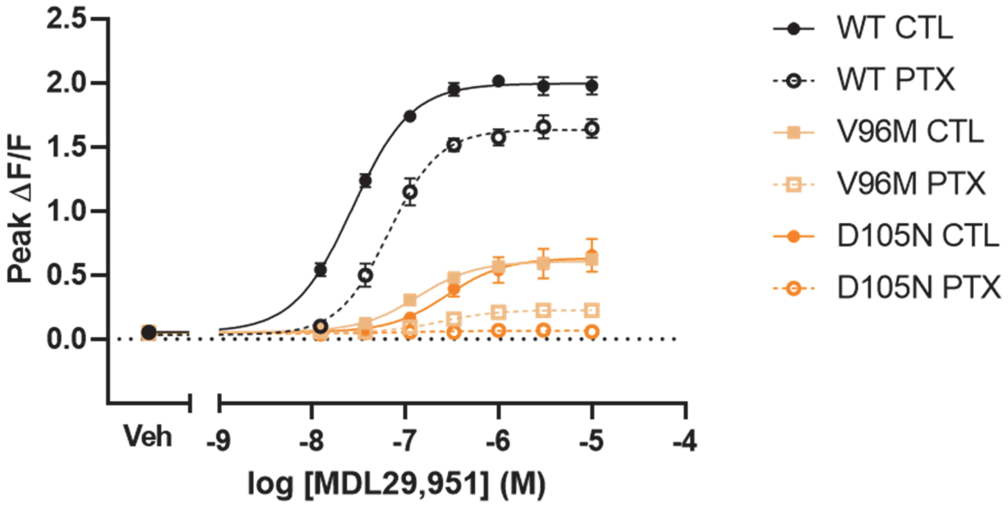

Figure S6. Gi/o-Gβγ subunits contributed to MDL29,951-stimulated calcium mobilization mediated by hGPR17L-WT, hGPR17L-V96M, and hGPR17L-D105N. HEK293 cells were transfected with hGPR17L-WT or -variants, pretreated with vehicle or 100 ng/mL pertussis toxin overnight, and calcium responses were subsequently measured upon treatment with MDL29,951. Data points represent mean±SEM of three independent experiments. CTL, vehicle control. PTX, pertussis toxin.

Figure S7

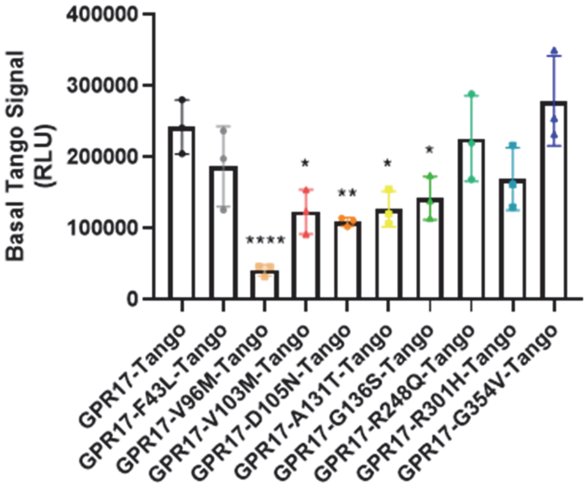

Figure S7. Human GPR17L-Tango variants have differential constitutive β-arrestin recruitment. Basal luminescence was measured in HTLA cells that were transiently transfected with hGPR17L-WT-Tango or hGPR17L-Tango variants. Data represent the basal (vehicle) levels of β-arrestin recruitment and were analyzed by one-way ANOVA with Dunnett's post hoc test compared to hGPR17L-WT-Tango. *, $p < 0.05$, **, $p < 0.01$, ****, $p < 0.0001$.

Figure S8

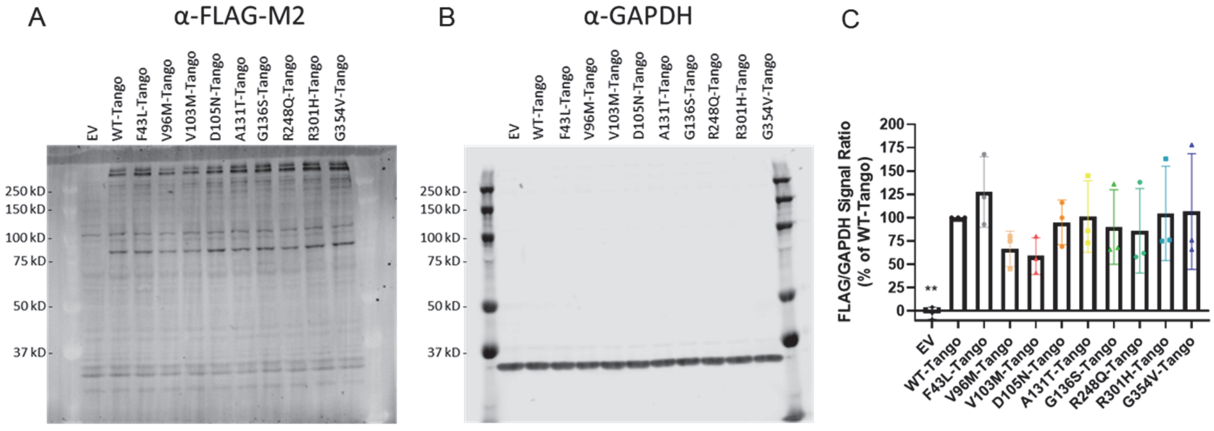

Figure S8. Human GPR17L-Tango variants have similar expression levels to hGPR17L-WT-Tango in HTLA cells. Representative western blots for **(A)** FLAG epitope-tagged hGPR17L-WT-Tango and hGPR17L-Tango variants and **(B)** GAPDH from HTLA cells that were transiently transfected with pcDNA3.1(-) empty vector, hGPR17L-WT-Tango, or hGPR17L-Tango variants. **(C)** Quantification of FLAG/GAPDH signal ratio for western blots of lysates from three independent transfections. The FLAG signal was the sum of hGPR17L-Tango-specific immunoreactive bands at ~85 kD and greater than 250 kD. Data represent mean±SD and were analyzed by one-sample t-test compared to 100. **, $p < 0.01$. EV, empty vector.

Figure S9

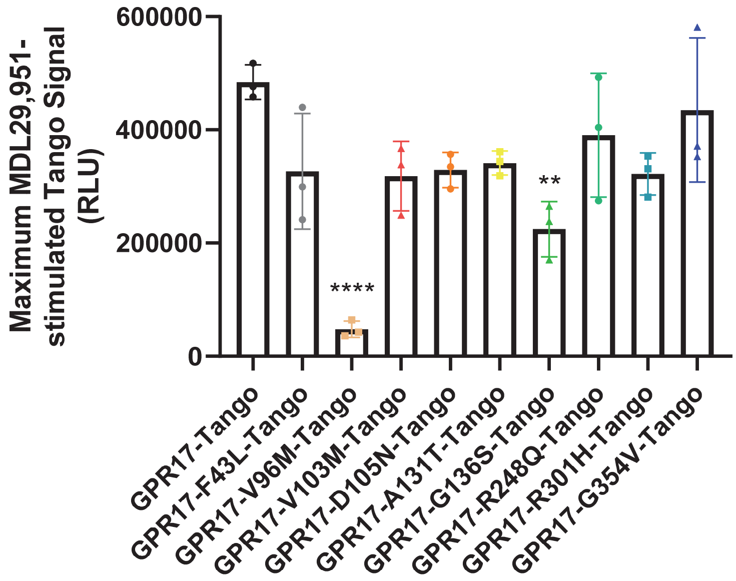

Figure S9. Human GPR17L-Tango variants V96M and G136S have impaired maximum agonist-stimulated β-arrestin recruitment as compared to hGPR17L-WT. HTLA cells were transiently transfected with hGPR17L-WT-Tango or hGPR17L-Tango variants and treated with MDL29,951 (ranging from 0.5 nM – 30 µM). The luminescence responses were measured from duplicate wells and data were fit to a sigmoidal dose-response equation. Data represent mean±SD for the maximum agonist-stimulated responses, in the form of the calculated top of the dose-response curve, from three independent experiments that were reported in Table 5 and Figure 6. Data were analyzed by one-way ANOVA with Dunnett's post hoc test compared to hGPR17L-WT-Tango. **, $p < 0.01$, ****, $p < 0.0001$.

Figure S10

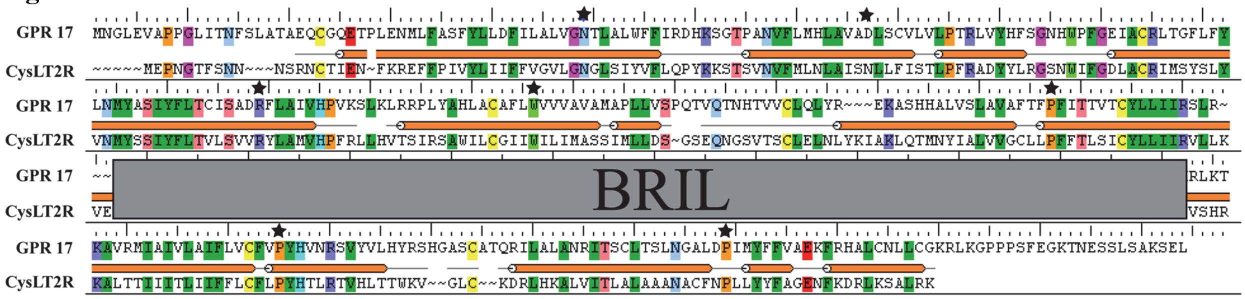

Figure S10. Sequence alignment of human CysLT2R and GPR17. The identical residues were highlighted and colored according to the properties of the amino acids. The highly conserved anchor residues in each TM domain were labeled with stars. The secondary structures are depicted as brown tubes between the sequences. The inserted residues of apocytochrome b_{562}RIL were boxed.
